# Transfer and transformation characteristics of Zn and Cd in soil-rotation plant (*Brassica napus* L and *Oryza sativa* L) system and its influencing factors

**DOI:** 10.1038/s41598-023-34377-4

**Published:** 2023-05-06

**Authors:** Qiuxiao Yan, Hui Fang, Daoping Wang, Xuefeng Xiao, Tingfei Deng, Xiangying Li, Fuxiao Wei, Jiming Liu, Changhu Lin

**Affiliations:** 1 Key Laboratory of Chemistry for Natural Products, Guizhou Medical University, Guiyang, China; 2 Natural Products Research Center of Guizhou Province, Guiyang, China; 3 College of Forestry, Guizhou University, Guiyang, China; 4 Beautiful Village Construction Center of Quzhou City, Quzhou, Zhejiang China; 5 Institute of New Rural Development, Guizhou University, Guiyang, China; 6 Department of Labor Health and Environmental Hygiene, School of Public Health, Guizhou Medical University, Guiyang, China

**Keywords:** Agroecology, Plant sciences, Environmental sciences

## Abstract

Rice–rape rotation is a widely practiced cropping system in China. However, changes in soil properties and management could change the bioavailability of Cd, In order to explore the occurrence state, transportation and transformation characteristics of heavy metals Cd and Zn in rice-rape rotation system in Guizhou karst area with high background value of Cd. In the karst rice–rape rotation area, the physical and chemical properties of soil, chemical specifications and activities of Cd and Zn at different soil depths and during various crop growth stages, and the bioaccumulation of Cd and Zn in different tissues of rice and rape were studied by field experiment and laboratory analysis. The bioaccumulation of Cd and Zn and the effects of physical and chemical soil properties on the activities and bioavailabilities of Cd and Zn during rice–rape rotation were explored. The findings revealed that soil particle size, composition, pH, redox potential, soil organic matter, and Cd and Zn contents varied dramatically, especially in deep soils. The physical and chemical properties of the deep and surface soils were significantly related to the bioaccumulation of Cd and Zn. Cd and Zn are activated during crop rotation. Cd was easier to be enriched in rice, while Zn was easier to be enriched in rape. The correlation between Cd and Zn contents in *Brassica campestris* L and their enrichment abilities were not significant, but that in *Oryza sativa* L were significant. During rice-rape rotation, the chemical speciations and activities of Cd and Zn changed with the changes of soil properties and waterlogging environment. This study had important basic guiding significance for the evaluation, prevention and control of heavy metal pollution, and improving soil quality in different rotation systems in karst areas, and was conducive to promoting the safe production of rape and rice.

## Introduction

The soil–plant system is the basic structural unit of the biosphere and the main object of soil environmental research^1^. As an important component of the environment and a precious renewable resource, soil can promote the development of human agricultural productivity; however, it can also be polluted by heavy metals caused by human activities and civilization. In recent years, with the rapid economic development, the environmental problems of the ecosystem have been amplified as a result of increasing heavy metal pollution of the soil caused by anthropogenic activities (such as mining, and industrial and agricultural activities)^2^. In China, excess rates of heavy metals were found in 19.4% of farmland soil, significantly higher than that in grasslands, forests, and other environmental types^3^. According to the Chinese Soil Environmental Quality Standard (CSEQS) GB 15618-2018, high geological background levels (unusually high levels of heavy metals in soils and their parent materials) are an important factor leading to excessive heavy metals in the soil^4–6^. Southwest China is an area with typically high geological background levels^1,7^. In Guizhou province, where carbonate rocks are widely distributed, the background heavy metal levels in the soil are generally high owing to the influence of topography and parent materials^8^. Cai et al.^9^ showed that the background value of Cd in cultivated soil in Guizhou Province was 0.10 ~ 1.93 mg/kg (average 0.40 mg/kg), and that of Zn was 49.77 ~ 218.19 mg/kg (average 104.21 mg/kg). Under natural conditions, the bioavailability of metals in such soil is not very high because of soil alkalinity or because the metal is combined with mineral structures, both circumstances resulting in a low uptake of metals by plants^1^. However, with the development of more intensive agricultural activities, soil’s physical–chemical proprieties are changed and the activity of heavy metals is gradually activated. Among them, human factors, such as crop rotation, exert a significant impact on the content of heavy metals in the soil. This impact leads to a high degree of local variation in the distribution of heavy metals^10^. In addition, heavy metals are characterized by their long incubation period and irreversibility, which means they can accumulate through the food chain even at low concentrations, posing a serious and persistent threat to humans, animals, and the environment^11,12^.

Rice (*Oryza sativa* L) plantations are widespread in south Asian countries and cover an estimated area of 26.7 million ha^13,14^. In China, approximately 72–86% of the country’s rice is cultivated using upland–paddy rotation systems, which cover approximately 13 million hectares^15,16^. Globally, rice contaminated with Cd has become one of the major human Cd exposure pathways. This issue is of great concern because rice is very important for food security^17^. Rape (*Brassica napus* L.) is the second largest source of edible oil in the world^18^, with high nutritional value and good fatty acid composition for both humans and animals^19^. Because of its short growth period, high biomass, and strong enrichment ability, it has great application potential for remediation of Cd-contaminated soil^20^. As a result of these factors, the global demand for rape has increased sharply. Rice–rape rotations are important in south Asian countries^13^. In the context of the continuous increase of population and continuous decrease of arable land, the water-drought rotation tillage mode plays a crucial role in improving grain yield and ensuring food security. It can not only rationally utilize soil nutrients and water, improve farmland utilization efficiency, but also increase grain and oil production and guarantee grain and oil security^16^. However, crop rotation alters the physical and chemical properties of the soil, activates heavy metals in carbonate soil, and increases their bioavailability^8^.

It is important to note that the total heavy metal concentrations in soil may not be entirely consistent with their bioavailability. This inconsistency is due to the directly influencing factor of chemical speciation of heavy metals. The bioavailability of heavy metals generally refers to the chemical species of heavy metals that can exert toxic effects on organisms or be absorbed by them^21^. Different chemical species of heavy metals produce varying levels of toxicity to plants. Tessier et al.^22^ divided heavy metals into five fractions: exchangeable fraction (EXC), carbonate bound fraction (CAR), iron–manganese oxide bound fraction (IMO), organic-matter-bound fraction (OM), and residual fraction (RES). EXC can be directly absorbed by plant roots, which is, therefore, the most direct and effective fraction. CAR and IMO can be released from soil under certain conditions and are potentially bioavailable fractions. In contrast, OM and RES are neither easily released into the environment nor easily absorbed by plants^23^. However, the bioavailability of heavy metals is affected by many factors in the soil environment, including pH, soil organic matter (SOM) content, redox potential (Eh), soil structure, and ion interaction^24–26^. The rice–rape rotation is a process of changing water environments. The alternating of wet and dry soil states changes soil aeration, microbial activity and diversity, and soil physical and chemical properties, thus affecting heavy metal chemical speciation and bioavailability in the soil^26,27^. Since flooded paddy fields provide anaerobic conditions, S^2+^ produced by the anaerobic decomposition of organic matter (e.g. reductant sulfur bacteria) easily form and precipitate cadmium sulfide (CdS)^28,29^. Additionally, the dissolution of iron–manganese oxides and organic matter changes the pH and Eh of the soil and then affects the precipitation–dissolution equilibrium system of Cd^30^. During this process, the speciation change and the activity of other metals may also alter the interaction between metal ions, thus affecting the biotoxic effects of heavy metals^31^. As an essential heavy metal utilized by both plants and humans, Zn exerts a dual effect on plant growth and human health and both insufficient and excessive intake can have adverse effects^31^. Zn may interact with Cd, and changes in the Zn/Cd ratio significantly affect the availability of Zn and Cd in the soil^32^. For example, Zn can effectively inhibit the absorption and transport of Cd in vegetables and other plants. This process significantly reduces the Cd content in the edible parts of crops, thereby inhibiting the absorption of Cd by animals^33,34^. However, the ecological effects of this interaction are influenced by the concentration of the metals and the crop species involved^35^. In the process of crop rotation, the physical and chemical properties of the soil are altered. The effects on the speciation fraction and interaction of Cd and Zn in the soil are also altered, thus affecting their bioavailability. Therefore, in this study, alterations in physical and chemical soil properties, contents, chemical species, and the activities of the heavy metals Cd and Zn in the soil–plant system during different growth stages of rice–rapeseed rotations were analyzed. Additionally, the effects of the physical and chemical properties of the soil on the migration, transformation, and bioconcentration of Cd and Zn in the soil were explored. The findings are expected to enhance our understanding of the changes in physical and chemical soil properties, Cd and Zn contents, and chemical speciation and activity involved in the process of drought–flood rotation in karst areas as well as the effects on the bioaccumulation of Cd and Zn in *B. napus* and *O. sativa* L. Furthermore, the obtained data are envisaged to provide the basis for an effective strategy for soil phytoremediation in karst regions.

## Materials and methods

### Experimental material varieties

The *B. napus* L. variety used was Youyan 57, which is a sweet blue type semi-winter recessive genic male sterile, two-line hybrid. Its national certification number is 2013001. The *O. sativa* L variety used was Yixiang 725, which was crossbred from Yixiang 1A and the self-selected restorer line Mianhui 725 by the Mianyang Institute of Agricultural Science.

### Field planting

The study area was located in the town of Shiban (1066 44′ 28″ E, 27o 30′ 11″ N), Bozhou District, Zunyi City, Guizhou Province, which has a subtropical monsoon humid climate with an average annual temperature of 14.6 °C–16 °C, an average annual precipitation 900–1 100 mm, a frost-free period of 250–280 days, and annual sunshine of 1 000–1 100 h. The terrain is hilly and includes an intermountain basin at an altitude of approximately 800 m, and the soil type is calcareous paddy soil. The experimental field area was approximately 450 m^2^, and there were no industrial or mining pollution sources or sewage irrigation infrastructure in the nearby area.

**Figure 1:**
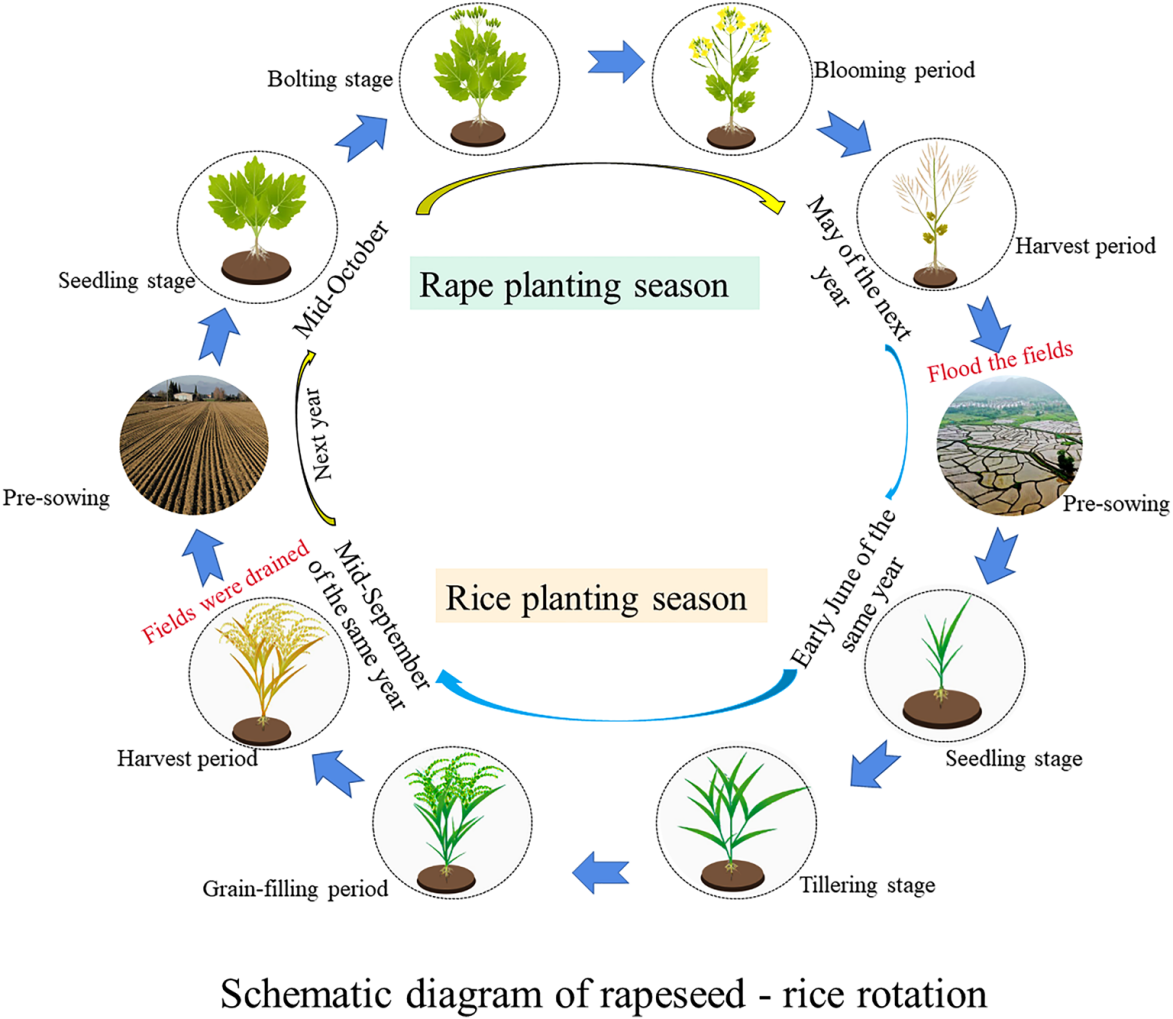
Schematic of rapeseed–rice rotation process.

The rape–rice rotation was designed according to the local traditional farming method: *B. napus* L was sown in mid-October and harvested at the end of May of the following year. The fields were filled with water for the transplantation of the *O. sativa* L seedlings after the *B. napus* L was harvested. The *O. sativa* L seedlings were raised in late April, transplanted in early June, and harvested in mid-September. The paddy fields were drained before the harvest of *O. sativa* L. After thinning, the row spacing of *O. sativa* L was 35 cm and the spacing of seedlings was 20 cm. This spacing was replicated for *B. napus* L (Fig. 1). The irrigation water was mountain streams with 0.001 mg/L Cd and 0.03 mg/L Zn content.

### Sample collection and preparation

Samples of the surface soil (0–20 cm depth) and deep soil (20–40 cm depth) were collected before sowing and during different growth stages of the two crops (*B. napus* L: seedling stage, bolting stage, blooming period, and harvest period; *O. sativa* L: seedling stage, tillering stage, grain-filling period, and harvest period). Each sampling unit had five sampling points, and each sampling point was in a circular area with a radius of about 50–60 cm. The sampling points avoided rice field ridges, roads, etc. The sampling method for deep soil during flooding was as follows: a 20–40 cm soil column was collected using a stainless steel drill, and the outer layer of the soil that had touched the metal drill material was removed. Simultaneously, plant samples were collected in each soil sampling unit according to the soil sampling position, with the plant sampling points corresponding to the individual soil sampling points. Each sampling point was fixed, and continuous sampling was carried out in different growth stages, thus avoiding the error caused by the change of sampling points in the whole rotation process.

After the plant samples were brought to the laboratory, the tissues (roots, stems, leaves, flowers, etc.) were treated separately. The plant samples were washed with deionized water, dried at 75 °C ± 2 °C and ground to 0.25 mm. After the soil samples were air-dried in a cool and ventilated place, grinded and sifted to 2 mm, and then a part of the sample was taken by quartering method and continued grinding until the sample was sieved to 0.25 mm for analysis of physicochemical properties.

### Index analysis

Soil particle size analyses were performed using the hydrometer method^36^, and the Kaczynski system was used to classify the soil particles as follows^37^: fine clay (< 0.001 mm), coarse clay (0.001–0.005 mm), fine silt (0.005–0.01 mm), coarse silt (0.01–0.05 mm), fine sand (0.05–0.25 mm), and coarse sand (0.25–1 mm).

Soil pH (soil-distilled water ratio of 1:2.5 w/v) and Eh (direct determination with platinum electrode) were measured using a mV/pH combined meter (Rex Electric Chemical PHS-3E, China)^36^. The SOM was digested by K_2_Cr_2_O_7_-H_2_SO_4_ and examined using FeSO_4_ titration^36^. For total metal analysis, the soil and plant samples were digested, respectively, using HNO_3_ + HF (10:5 v/v) and HNO_3_ + H_2_O_2_ (10:5 v/v) and deionized water was maintained at a constant volume of 50 mL^36^. The Cd level was determined using a graphite furnace atomic absorption spectrometer (Agilent 240 Z, Agilent, Santa Clara, CA, USA), and Zn was determined using an inductively coupled plasma emission spectrometer (Leeman Prodigy XP, USA). Chemical speciation of Cd and Zn was determined using Tessier`s five-stage sequential extraction procedure^22^, and the supernatant extracted using the four-step method was tested using the same total metal analysis method. The content of RES was calculated using the subtraction method:

1 $$\mathrm{RES}={C}_{total\, metal}-\sum {C}_{i},$$

where* C*_*total metal*_ represents the total metal content, and *C*_*i*_ represents the contents of chemical speciation fractions (EXC, CAR, IMO, and OM, respectively).

Accuracy was assessed using the sample spike and recovery method, and the average recoveries of Cd and Zn were 91.2% and 102.6%, respectively. The ratio of the sum of the five chemical speciation fraction concentrations of heavy metals in the soil samples to their total concentrations ranged from 78.63 to 125.84%.

The activities of heavy metals in the soil can be described using their movement factor (*MF*)^38^:

2 $${MF}_{i}\left(\%\right)=\frac{{C}_{EXC}+{C}_{CAR}}{\sum {C}_{five \,speciation}}\times\, 100\%,$$

where, C_*EXC*_ and C_*CAR*_ represent the concentrations of EXC and CAR, respectively, and ∑*C*_*five speciation*_ represents the sum of the five chemical speciation fraction (EXC, CAR, IMO, OM, and RES) concentrations of the metal.

The activation degrees of soil heavy metals were expressed using the activation coefficient (*AD*):

3 $$AD=\frac{{MF}_{soil-rice}}{{MF}_{soil-rape}},$$

where, *MF*_*soil-rice*_ and *MF*_*soil-rape*_ represent the average movement parameters of heavy metals in the soil throughout the rape and rice planting seasons, respectively. *AD* > 1 indicates that soil heavy metals are activated, whereas *AD* < 1 indicates that soil heavy metals are passivated.

The bioconcentration factor (*BCF*) is the ratio of heavy metal content in plants to that in soil^7^. *BCF* represents the degree of migration of heavy metal elements in soil–plant systems and the heavy metal enrichment capacity of plants^1^. *BCF* is expressed using the following formula:

4 $$BCF=\frac{{C}_{i}}{{C}_{soil}},$$

5 $$T-BCF=\frac{{C}_{plant}}{{C}_{soil}},$$

where, *BCF* and *T*-*BCF* represent the bioenrichment capacities of different plant tissues and whole plants toward soil heavy metals, respectively. The *C*_*i*_,* C*_*plant*_, *and C*_*soil*_ represent the contents of heavy metals in different aboveground tissue parts, whole plants of *B. napus L* and *O. sativa* L, and soil, respectively.

The biotranslocation factor (*BTF*) is an evaluation of the ability of plant tissues to transport heavy metals^39^:

6 $${BTF}_{stem}=\frac{{C}_{stem}}{{C}_{root}},$$

7 $${BTF}_{other \,tissues}=\frac{{C}_{other\, tissues}}{{C}_{stem}},$$

8 $${BTF}_{rape\, pod \,to \,rapeseed}=\frac{{C}_{rapeseed}}{{C}_{rape\, pod}},$$

9 $${BTF}_{rice\, husk \,to\, rice}=\frac{{C}_{rice}}{{C}_{rice\, husk}},$$

10 $$T-BCT=\frac{\sum {C}_{above-ground\, parts}}{{C}_{root}},$$

where, *C*_*root*_, *C*_*stem*_, *C*_*rapeseed*_, *C*_*rape pod*_, *C*_*rice husk*_, and *C*_*rice*_ represent the heavy metal contents of the roots and stems of *B. napus* L or *O. sativa* L, rapeseed, rape pod, rice husk, and rice, respectively; *C*_*other tissues*_ represents the heavy metal contents of the leaves, followers, rape pod, and rapeseed of *B. napus* L or the leaves, rice in the husk, rice husk, and rice of *O. sativa* L; and *C*_*aboveground parts*_ represents the aboveground part of *B. napus* L or *O. sativa* L.

### Statistical analysis

All data have been expressed as means and standard errors of three replicates. The data were subjected to analysis of variance using the SPSS, ver.21.0 statistical software package (SPSS, Chicago, IL, USA), and differences between the mean values were compared using the least significant difference post-hoc test. *P* values of < 0.05 were considered statistically significant. Correlation coefficients between the variables were tested using Pearson’s correlation. Principal component analysis (PCA) and stepwise regression equations were used to evaluate the influencing factors of soil heavy metal activity and plant bioaccumulation. Graphs were constructed using OriginPro 9.0 (Origin Lab, Northampton, MA, USA).

### Ethics statement

The collection of plant materials in this study complied with relevant institutional, national, and international guidelines and legislation.

## Results

### Soil properties

#### Soil particle size composition

**Figure 2:**
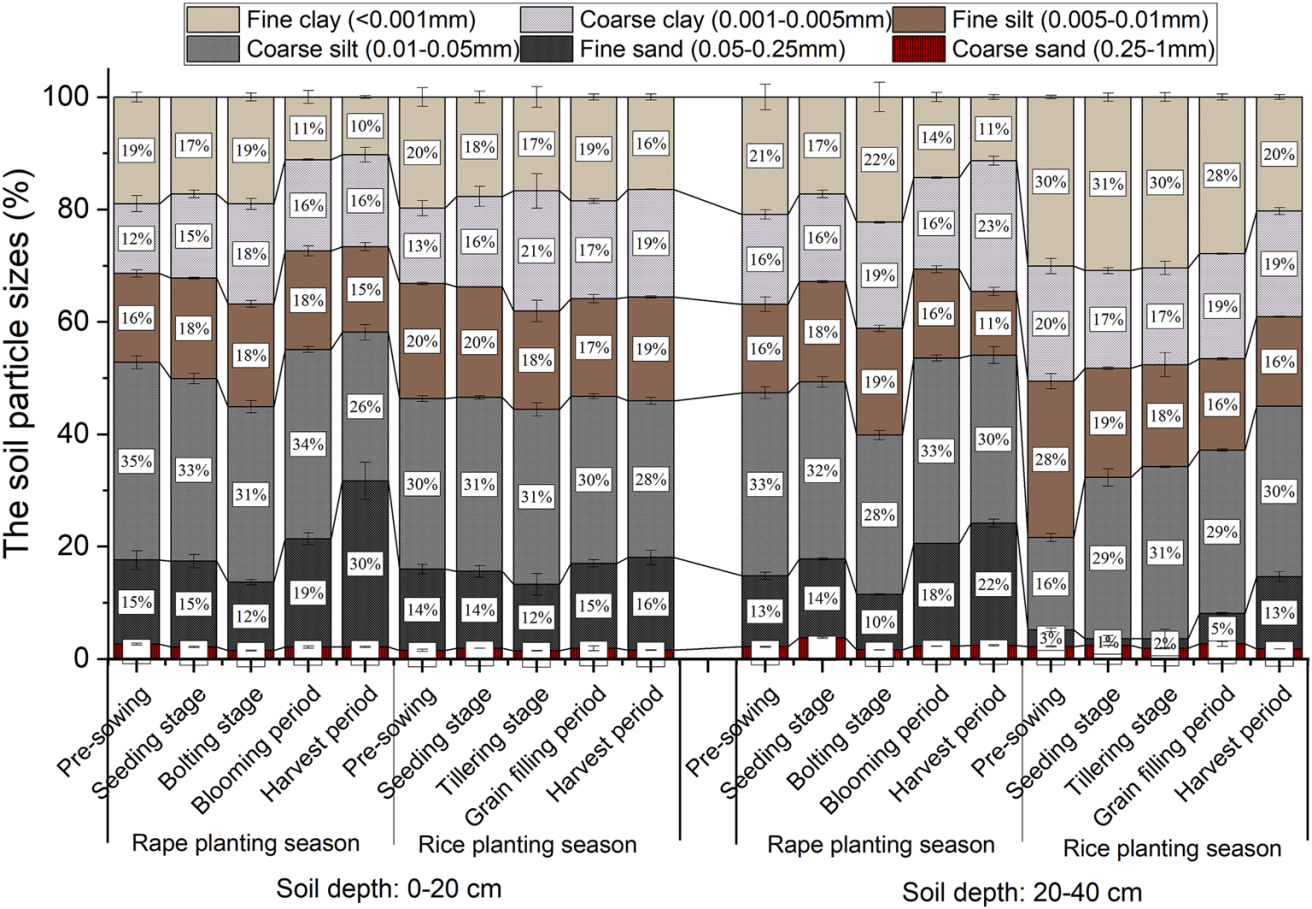
Changes of different depth soil machinery during rape–rice rotation. All values were mean of three replicates (n = 3), vertical bars represent the standard errors of the means based on three replicates.

As shown in Fig. 2, in surface soil (0–20 cm depth), the percentages of coarse silt (26.43–35.21%) were the greatest (except during the rape harvest stage) and those of coarse sand (1.46–2.63%) were the smallest. In deep soil (20–40 cm depth), coarse silt contents were the greatest (16.43–34.09%), followed by fine clay content (10.54–30.89%). Overall, compared with surface soil, deep soil was more viscous, and the percentages of clay particles in the soil during the rice planting season were higher than those during the rape planting season. These differences were more obvious in deep soil (Fig. 2). In addition, the variation coefficients (*cv*) of the percentages of fine sand and fine clay were 31.41% and 18.88%, respectively, in surface soil (Table 1). However, in deep soil, fine sand (*cv* = 68.90%) and fine clay (*cv* = 30.01%) showed strong variability, followed by coarse sand (*cv* = 24.39%) and fine silt (*cv* = 23.26%) (Table 1). These results showed that after soil flooding, clay particles, fine silt particles, and other materials move vertically along the soil pores under the action of water gravity, causing the clay particles of paddy soil to move down, and the core soil layer is relatively viscous.

**Table 1:** Coefficient of variation of soil particle size composition.

Soil depth	Coefficient of variation (*cv*) (%)					
	Fine clay (< 0.001 mm)	Coarse clay (0.001–0.005 mm)	Fine silt (0.005–0.01 mm)	Coarse silt (0.01–0.05 mm)	Fine sand (0.05–0.25 mm)	Coarse sand (0.25–1 mm)
0–20 cm	18.88	15.94	8.44	7.94	31.41	18.88
20–40 cm	30.01	10.42	23.26	15.91	68.90	24.39

All values are mean of three replicates (n = 3).

#### Soil pH

**Figure 3:**
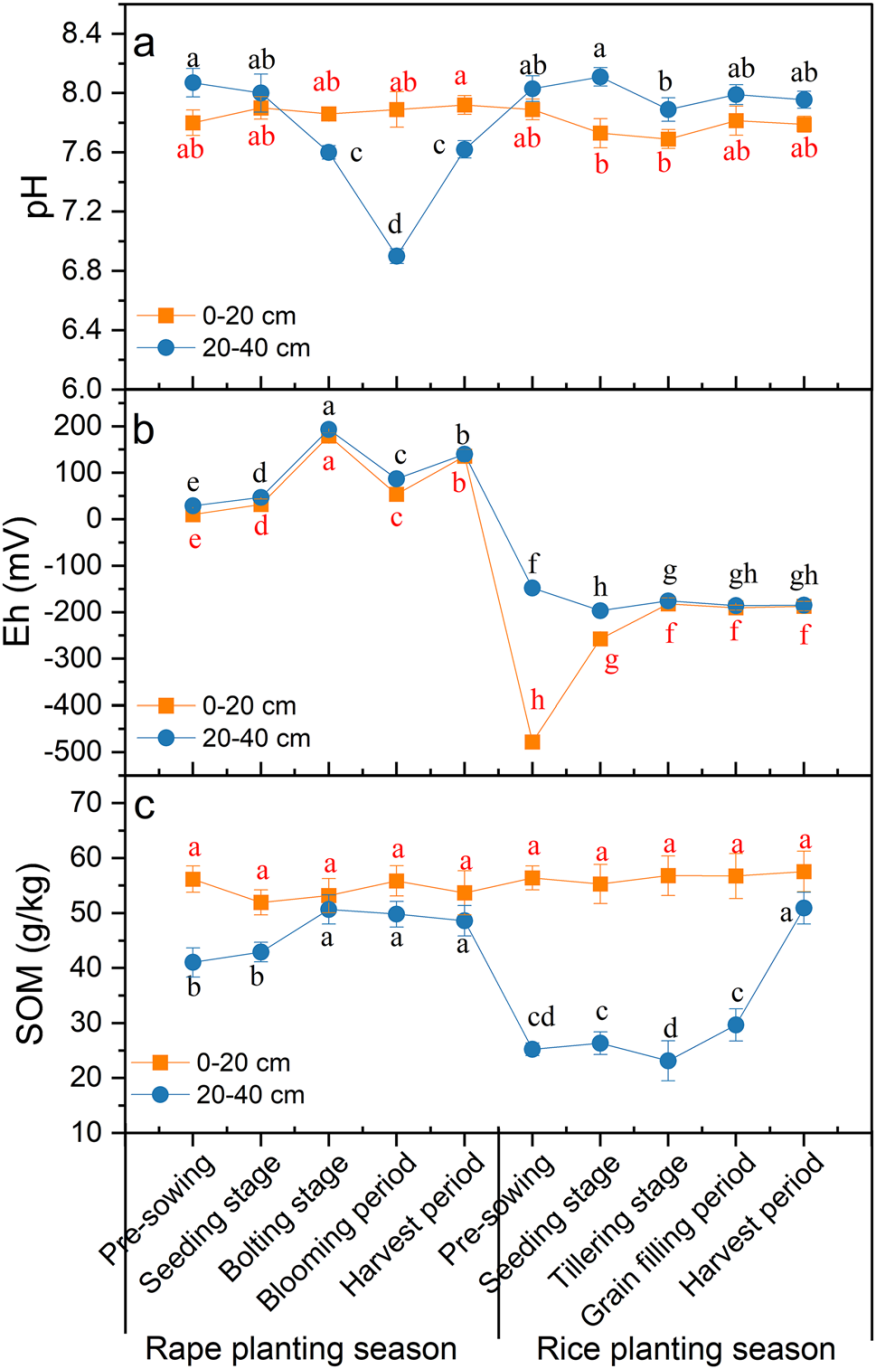
Changes in the pH (**a**), Eh (**b**) and SOM (**c**) of the different depths soil during rape–rice rotation. Vertical bars represent the standard errors of the means based on three replicates, and different lowercase letters indicate that the same layer of soil varies significantly at different stages (LSD test; *P* < 0.05).

The soil in the study area was neutral to alkaline (Fig. 3a), which is typical for calcareous soil developed from carbonate rock. Throughout the rape planting season, the pH value of the surface soil (Fig. 3a) did not change significantly and varied within a very narrow range (7.80–7.92). On the contrary, the pH value of deep soil changed significantly (6.9–8.07) and showed a downward trend that began during the pre-sowing period and extended into the blooming period. The pH rose again during the harvest period (Fig. 3a). Throughout the rice planting season, the trends in pH change were the same in surface and deep soil; in deep soil, it was higher (7.89–8.11) than that in surface soil (7.69–7.89) (Fig. 3a). In general, the change of soil pH in deep soil was greater than that in surface soil throughout the rape and rice planting season (Fig. 3a).

#### Soil Eh

The Eh of the soil was significantly affected by water level, soil depth, and the interactions of these factors (Fig. 3b). Eh values were positive throughout the rape planting season, and the Eh of surface soil (10.20–179.30 mV) and deep soil (29.2–193.3 mV) rose in a fluctuating pattern (Fig. 3b). While the Eh of surface soil and deep soil decreased sharply to negative values after rape harvest, the Eh of surface soil increased gradually during the rice seedling stage and did not differ much from that of deep soil at the tillering stage. Finally, both tended to be stable (Fig. 3b), and overall, an obvious variation was observed in the Eh of the soil during the flooding and drainage periods. Additionally, the Eh variations of surface soil were greater than those of deep soil (Fig. 3b).

#### SOM content

The SOM content was clearly affected by the alternation of drought and flood (Fig. 3c). The SOM of surface soil showed no significant change and was higher than that of deep soil, whereas the change in deep soil was obvious, especially during the rice planting season. The SOM content of deep soil before sowing rice decreased by 23.37 g/kg compared with that during the rape harvest period but increased slightly after the tillering stage (Fig. 3c).

### Cd and Zn contents in the soil

**Figure 4:**
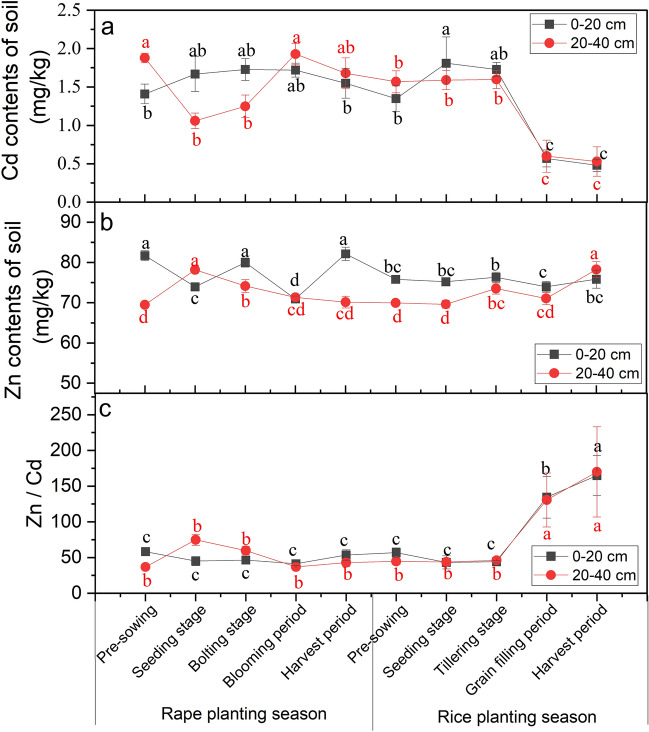
Changes in the contents of Cd (**a**) and Zn (**b**), and the ratio of Zn to Cd content (Zn/Cd) (**c**) in the different depth soil (0–20 cm and 20–40 cm) during rape–rice rotation. Vertical bars represent the standard errors of the means based on three replicates, and different lowercase letters indicate that the same layer of soil varies significantly at different stages (LSD test; *P* < 0.05).

**Figure 5:**
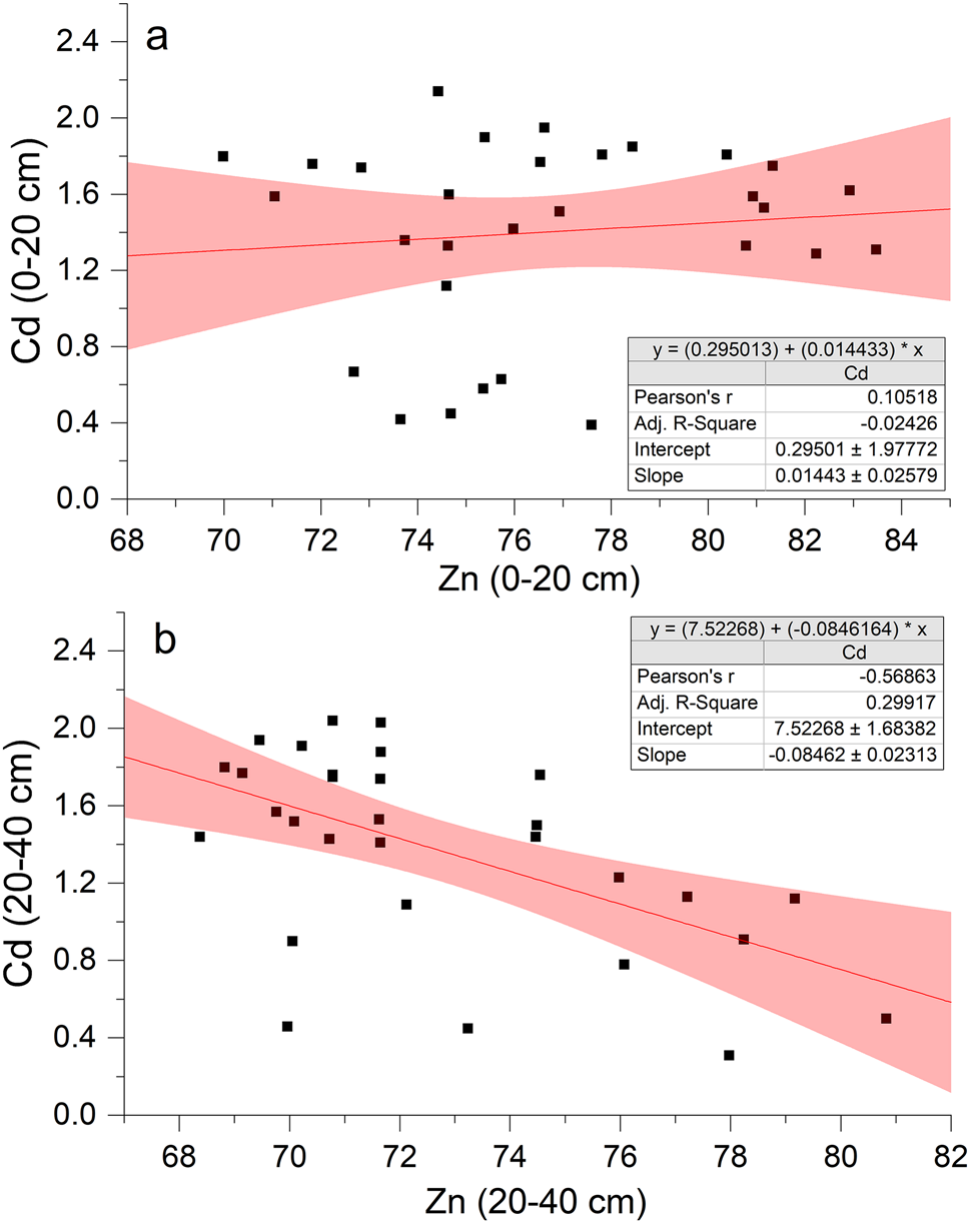
Linear relationship between Cd and Zn contents in surface soil (0–20 cm) (**a**) and deep soil (20–40 cm) (**b**).

As shown in Fig. 4a, Cd contents in both surface (0.48 mg/kg–1.81 mg/kg) and deep soil (0.53 mg/kg–1.93 mg/kg) first increased and then decreased throughout the rape and rice planting season (except during rape pre-sowing). The content of Zn in surface soil (69.98 mg/kg–83.47 mg/kg) was higher than that in deep soil (68.37 mg/kg–80.82 mg/kg) and fluctuated greatly in surface soil during the rape planting season (Fig. 4b). The Zn/Cd ratio (Fig. 4c) showed little variation throughout the rape planting season until the rice tillering stage but increased significantly after that. There was no significant correlation between Cd and Zn in surface soil (Fig. 5a), but there was a significant negative correlation (*r* =  − 0.569) in deep soil (Fig. 5b), indicating that Cd and Zn had obvious interaction inhibition (Fig. 5b).

### Chemical speciation of Cd and Zn in the soil

**Figure 6:**
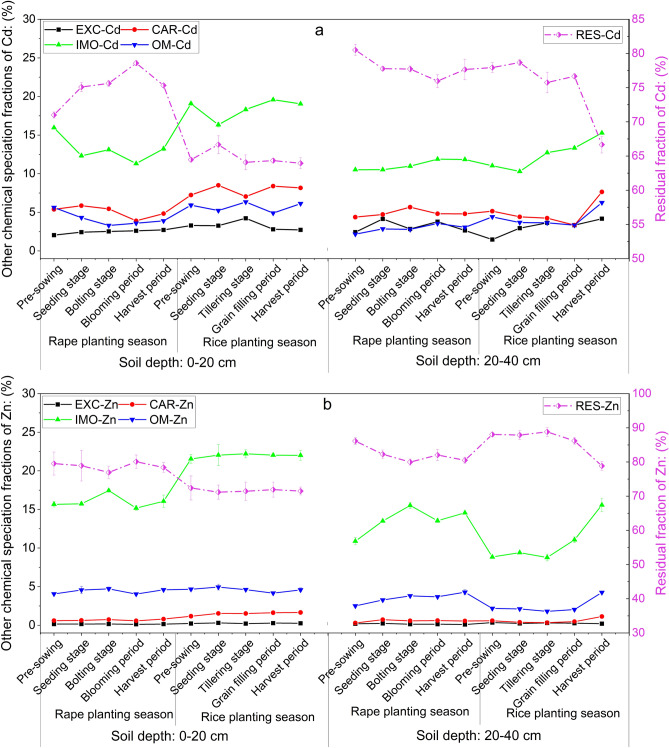
Changes of chemical speciation fractions of Cd (**a**) and Zn (**b**) in soils at different depths (0–20 cm and 20–40 cm). The five chemical speciation fractions were exchangeable fraction (EXC), carbonate bound fraction (CAR), iron–manganese oxide bound fraction (IMO), organic-matter-bound fraction (OM), and residual fraction (RES). Vertical bars represent the standard errors of the means based on three replicates.

The proportions of Cd chemical speciation fractions generally followed the sequence RES-Cd > IMO-Cd > CAR-Cd > OM-Cd > EXC-Cd (Fig. 6a) and Zn followed the sequence RES-Zn > IMO-Zn > OM-Zn > CAR-Zn > EXC-Zn (Fig. 6b). The changes in chemical speciation fractions of Cd and Zn were obvious during the rape–rice rotation. The five chemical speciation fractions of Cd in the surface soil fluctuated greatly, but the fluctuations of Zn were smaller. The fluctuations of Cd in deep soil were also smaller than those in surface soil, but those of Zn were greater.

**Figure 7:**
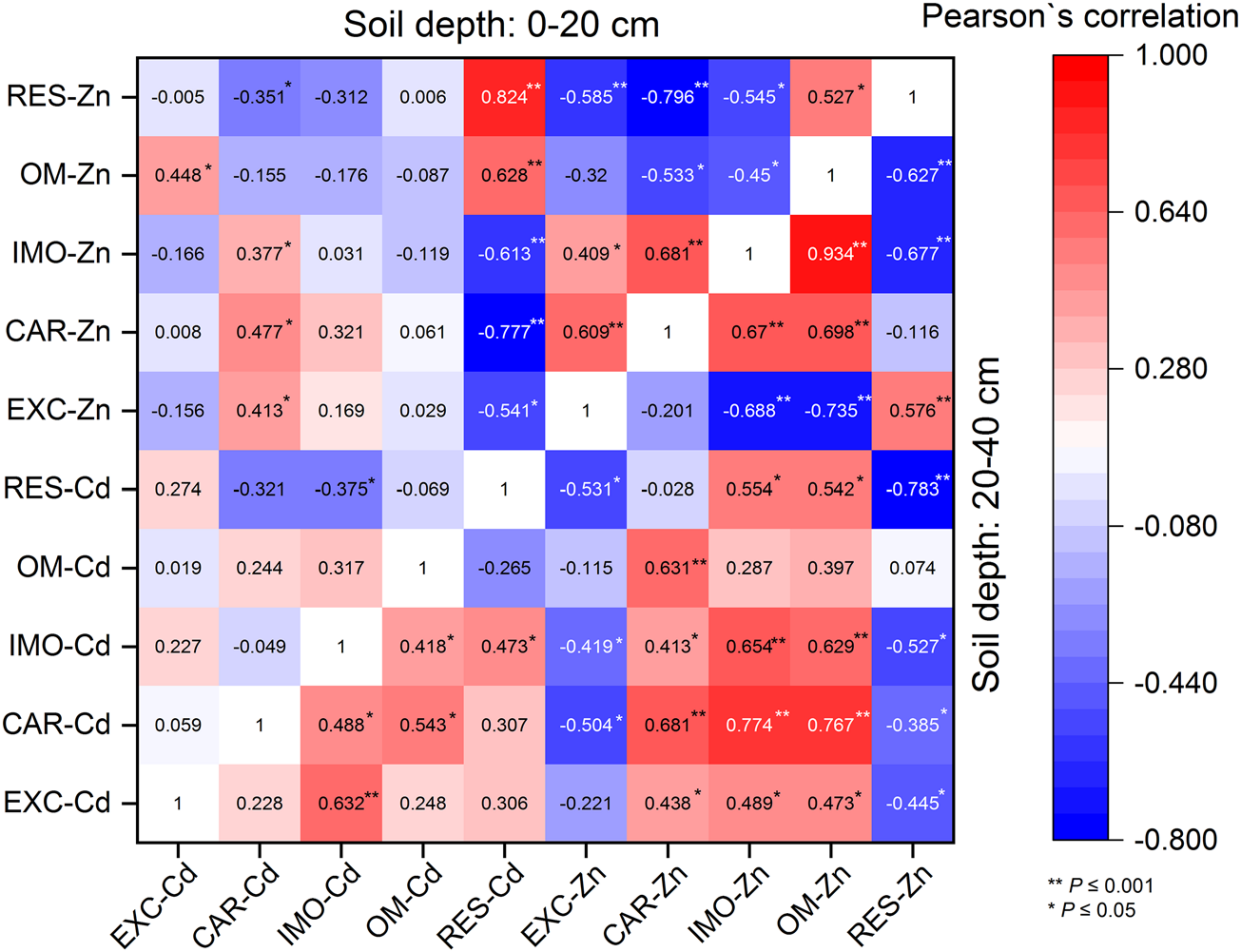
Pearson’s correlation coefficients among exchangeable fraction (EXC), carbonate bound fraction (CAR), iron–manganese oxide bound fraction (IMO), organic-matter-bound fraction (OM), and residual fraction (RES) of Cd and Zn in soils at different depths (0–20 cm and 20–40 cm).

In surface soil, the percentages of EXC-Cd, CAR-Cd, OM-Cd, and IMO-Cd during the rice planting season were higher than those during the rape planting season (increasing by an average of 32.49%, 54.79%, 40.06%, and 37.7%, respectively), whereas the percentages of RES-Cd were the opposite (decreased by 13.88%), and among CAR-Cd, OM-Cd, and IMO-Cd, they mostly decreased during the rape planting season and increased during the rice planting season. Moreover, RES-Cd was negatively correlated with IMO-Cd (*r* =  − 0.375) and CAR-Cd (*r* =  − 0.321). Meanwhile, OM-Cd was positively correlated with IMO-Cd (*r* = 0.317) and negatively correlated with RES-Cd (*r* =  − 0.069) (Fig. 7). The results also showed that the chemical speciation of Cd in the surface soil changed from RES-Cd to CAR-Cd, IMO-Cd, OM-Cd, and EXC-Cd. However, compared with surface soil, the percentages of the five chemical speciation fractions of Cd in deep soil varied to a lesser extent (Fig. 6a). Moreover, it is noteworthy that the percentages of RES-Cd and RES-Zn decreased significantly from the grain-filling period, whereas those of the other four fractions increased significantly (Fig. 6a,b).

Similar to Cd, the proportions of EXC-Zn, CAR-Zn, IMO-Zn, and OM-Zn in surface soil increased during the rice planting season compared with those during the rape planting season (increasing by an average of 74.30%, 125.41%, 37.18%, and 4.51%, respectively) (Fig. 6b), whereas the proportion of RES-Zn decreased by 9.00% (Fig. 6b). Likewise, the proportions of EXC-Zn and CRA-Zn in deep soil increased by 69.51% and 5.38%, respectively, whereas the changes in IMO-Zn and OM-Zn were opposite to those in surface soil (decreasing by 21.11% and 29.50%, respectively) (Fig. 6b). There were significant correlations among the chemical speciation fractions of Zn (Fig. 7), among which EXC-Zn, CAR-Zn, and IMO-Zn in the surface soil showed significant positive correlations and the three fractions (EXC-Zn, CAR-Zn, and IMO-Zn) showed significant negative correlations with OM-Zn and RES-Zn. Additionally, there was a positive correlation between OM-Zn and RES-Zn (*r* = 0.527) (Fig. 7). On the contrary, in deep soil, EXC-Zn and RES-Zn were negatively correlated with the other three fractions, and there were significant positive correlations between EXC-Zn and RES-Zn (*r* = 0.576) and among CAR-Zn, IMO-Zn, and OM-Zn (*r* = 0.670, 0.698, and 0.934, respectively) (Fig. 7).

### Activation of the heavy metals Cd and Zn in the soil

**Figure 8:**
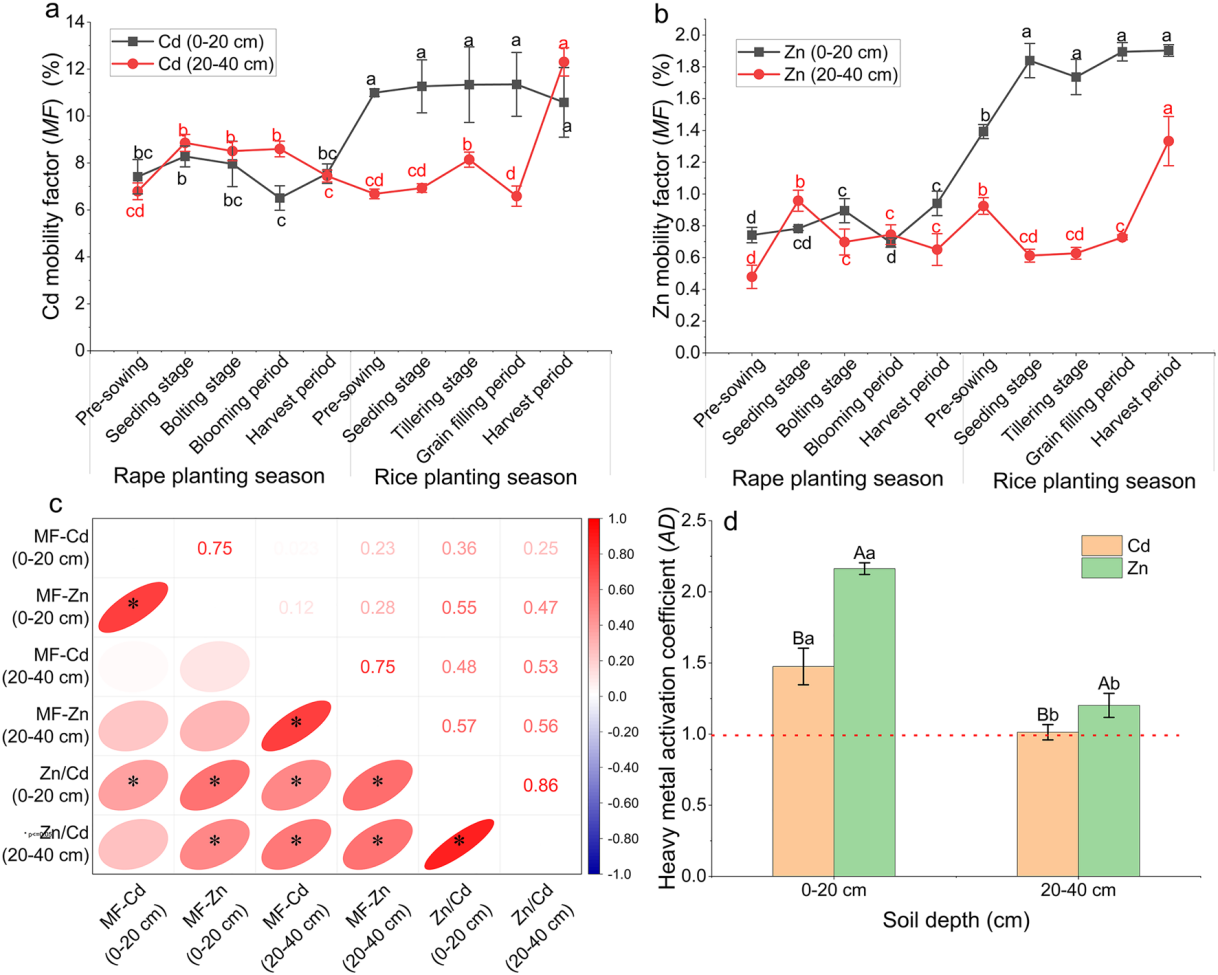
Changes in the mobility factor (*MF*) of Cd and Zn in soils at different depths during rape–rice rotation.(**a,b**); Pearson`s correlation analysis between mobility factors of Cd and Zn (*MF*-Cd, *MF*-Zn) and the ratios of Zn to Cd content (Zn/Cd) in soils at different depths during rape–rice rotation (**c**); and heavy metals activation coefficients (*AD*) in soils at different depths from rape planting season to rice planting season (**d**). Vertical bars represent the standard errors of the means based on three replicates. (**a,b**): Different lowercase letters indicate that the same layer of soil varies significantly at different stages (LSD test; *P* < 0.05); (**d**) Different lower case letters indicate significantly differences between different depths soil under the same heavy metal, and different capital letters indicate significant differences between different heavy metals under the same depths soil (LSD test; *P* < 0.05). (**c**) * indicate significant at *P* < 0.05.

Although the content of Zn in the soil was significantly higher than that of Cd (Fig. 4a,b) throughout the rape and rice planting season, the activity changes of Cd and Zn in surface soil and deep soil were not consistent. Specifically, the activities of Cd and Zn in surface soil increased significantly during the flooding process after the rape harvest and then remained stable during the rice planting season, which increased to 36.06–74.40% (Cd, Fig. 8a) and 87.75–172.79% (Zn, Fig. 8b). On the contrary, the activities in deep soil fluctuated slightly before the grain-filling period and then greatly increased (increased by 65.28% (Cd) and 104.90% (Zn)) (Fig. 8a,b). In the dry–wet alternating process from rape planting season to rice planting season, the activation coefficients (*AD*) of the heavy metals Cd and Zn were both > 1 and were activated to varying degrees. The activation effect of Zn was slightly higher than that of Cd (Fig. 8d), but the activity of Cd in the soil was significantly higher than that of Zn (Fig. 8a,b). This result further indicated that the activities of Cd and Zn were jointly expressed by their total contents and proportion of available chemical speciation fractions. Additionally, coexisting ions influenced each other's reactivity. Significant positive correlations were observed between the activities of Cd and Zn, both in surface soil and deep soil, and the change of the Zn/Cd ratio in the soil demonstrated significant positive correlations with the change of Cd and Zn activities (Fig. 8c). Furthermore, the alternating drought and flood cycles exhibited synergistic effects on the activation of Cd and Zn.

### Correlation analysis between the physical and chemical properties of the soil and the chemical speciation of heavy metals

As shown in Table 2, throughout the rape–rice planting season, coarse sand was significantly negatively correlated with the total content of Cd in both surface (*r* =  − 0.547) and deep soils (*r* =  − 0.700) and Eh exerted positive effects on the total contents of Cd and Zn, which were significant in surface soil. In deep soil, Cd content was significantly positively correlated with fine sand (*r* = 0.424) and Zn content was significantly positively correlated with SOM (*r* = 0.401).

**Table 2:** Pearson’s correlation coefficients of soil physical and chemical properties with the contents and five chemical speciation fractions of Cd and Zn.

Soil depth	Physical and chemical indexes of soil	Heavy metal content and chemical speciation states											
		Cd	EXC-Cd	CAR-Cd	IMO-Cd	OM-Cd	RES-Cd	Zn	EXC-Zn	CAR-Zn	IMO-Zn	OM-Zn	RES-Zn
0–20 cm	pH	0.100	0.036	− 0.315	− 0.143	− 0.062	0.587*	0.103	− 0.349	− 0.382*	− 0.327	0.408*	0.559*
	Eh	0.368*	0.031	− 0.443*	− 0.354	− 0.241	0.811*	0.401*	− 0.608*	− 0.777*	− 0.605*	0.596*	0.802*
	SOM	− 0.252	− 0.066	− 0.075	0.248	0.189	− 0.257	− 0.120	0.315	0.296	0.124	− 0.227	− 0.240
	Fine clay (< 0.001 mm)	− 0.011	− 0.225	0.168	0.222	− 0.014	− 0.316	0.090	0.287	0.063	0.066	− 0.169	− 0.290
	Coarse clay (0.001–0.005 mm)	− 0.098	0.203	0.158	− 0.102	− 0.033	− 0.176	− 0.174	0.261	0.469*	0.245	− 0.154	− 0.290
	Fine silt (0.005–0.01 mm)	0.005	− 0.036	0.210	0.216	− 0.049	− 0.267	− 0.300	0.228	0.189	0.335	− 0.171	− 0.482*
	Coarse silt (0.01–0.05 mm)	0.302	0.071	− 0.174	− 0.195	0.157	0.205	− 0.124	− 0.315	− 0.380*	− 0.422*	0.051	0.292
	Fine sand (0.05–0.25 mm)	0.276	0.063	− 0.409*	− 0.289	− 0.107	0.700*	0.323	− 0.576*	− 0.658*	− 0.478*	0.530*	0.744*
	Coarse sand (0.25–1 mm)	− 0.547*	− 0.069	0.480*	0.391*	0.127	− 0.826*	− 0.330	0.680*	0.911*	0.710*	− 0.619*	− 0.834*
20–40 cm	pH	− 0.335	− 0.512*	− 0.459*	− 0.605*	− 0.337	− 0.657*	0.031	0.469*	− 0.279	− 0.564*	− 0.616*	− 0.687*
	Eh	0.352	0.261	0.502*	0.376*	− 0.154	0.808*	0.058	− 0.650*	0.178	0.760*	0.725*	0.829*
	SOM	− 0.064	0.517*	0.726*	0.592*	0.296	0.476*	0.416*	− 0.640*	0.722*	0.936*	0.929*	0.679*
	Fine clay (< 0.001 mm)	− 0.197	− 0.457*	− 0.501*	− 0.471*	− 0.138	− 0.551*	− 0.195	0.604*	− 0.466*	− 0.731*	− 0.782*	− 0.719*
	Coarse clay (0.001–0.005 mm)	− 0.355	− 0.153	0.014	0.043	0.353	− 0.230	− 0.027	0.202	0.092	− 0.171	− 0.131	− 0.441*
	Fine silt (0.005–0.01 mm)	0.199	− 0.476*	− 0.205	− 0.439*	0.054	− 0.349	− 0.201	0.494*	− 0.164	− 0.453*	− 0.383*	− 0.438*
	Coarse silt (0.01–0.05 mm)	− 0.007	0.506*	0.211	0.212	− 0.215	0.366*	0.250	− 0.349	0.064	0.389*	0.266	0.540*
	Fine sand (0.05–0.25 mm)	0.424*	0.239	0.252	0.322	− 0.096	0.599*	− 0.058	− 0.547*	0.148	0.519*	0.581*	0.657*
	Coarse sand (0.25–1 mm)	− 0.700*	0.181	0.281	0.244	0.596*	− 0.499*	0.453*	0.067	0.633*	0.165	0.196	− 0.301

“*” indicate significant at *P* < 0.05.

In surface soil, there was no obvious correlation between physical and chemical properties or with EXC-Cd and OM-Cd (Table 2). Eh (*r* =  − 0.443) and fine sand (*r* =  − 0.409) content had significant negative effects on CAR-Cd but had significant positive effects on RES-Cd. Coarse sand had significant positive effects on CAR-Cd (*r* = 0.480) and IMO-Cd (*r* = 0.391) but had a significant negative correlation with RES-Cd (*r* =  − 0.826). pH had positive effects on RES-Cd (*r* = 0.587), OM-Zn (*r* = 0.408), and RES-Zn (*r* = 0.559) but was negatively correlated with CAR-Zn (*r* = − 0.382). EXC-Zn, CAR-Zn, and IMO-Zn were negatively correlated with fine sand and coarse silt but were significantly positively correlated with coarse sand (Table 2). In contrast, OM-Zn and RES-Zn were significantly positively correlated with fine sand and negatively correlated with coarse sand (Table 2).

In deep soil, except for the total content of Zn and EXC-Zn, pH was negatively correlated with the total content of Cd (*r* =  − 0.335) and other chemical speciation fractions of Cd, CAR-Zn, IMO-Zn, OM-Zn, and RES-Zn (Table 2). Eh levels were negatively correlated with OM-Cd (*r* =  − 0.154) and EXC-Zn (*r* =  − 0.650) but were positively correlated with other chemical speciation fractions of Cd and Zn. SOM was significantly negatively correlated with EXC-Zn (*r* =  − 0.640) but positively correlated with the chemical speciation fractions of Cd and Zn, all of which were significant, except for OM-Cd (Table 2). Besides the significant positive correlation with EXC-Zn (*r* = 0.604), fine clay was negatively correlated with Cd, Zn, and their chemical speciation fractions. Fine silt had a significant positive correlation with EXC-Zn (*r* = 0.494) and significant negative correlations with EXC-Cd, IMO-Cd, IMO-Zn, OM-Zn, and RES-Zn. Coarse silt had significant positive correlations with EXC-Cd, RES-Cd, IMO-Zn, and RES-Zn. Fine sand was positively correlated with RES-Cd, IMO-Zn, OM-Zn, and RES-Zn but negatively correlated with EXC-Zn. Coarse sand was negatively correlated with RES-Cd and positively correlated with OM-Cd and CAR-Zn.

Soil is a complex system, and its chemical properties frequently interact with each other to jointly affect the chemical speciation fractions of heavy metals in it. The activities of heavy metals are directly reflected by their total content and chemical speciation fractions. Step-up linear regression analyses indicated that CAR was the primary factor affecting the activity of Cd and Zn, followed by EXC, both of which had positive effects on the activity of Cd and Zn (*MF*-Cd and *MF*-Zn) (Table 3). On the contrary, Eh was the primary soil chemical property limiting the activities of heavy metals both in surface and deep soil (Table 3). Additionally, *MF*-Cd and *MF*-Zn were limited by their residual fractions in the surface soil. While *MF*-Cd was limited by fine silt, fine sand had a positive effect on it (Table 3). Meanwhile, pH and fine silt in deep soil had positive effects on *MF*-Cd and *MF*-Zn (Table 3), SOM had a positive effect on *MF*-Cd, and fine sand had a positive effect on *MF*-Zn (Table 3). However, *MF*-Cd was limited by IMO-Cd, and *MF*-Zn was limited by RES-Zn (Table 3).

**Table 3:** The influencing factors of movement factors of Cd and Zn (*MF*-Cd, *MF*-Zn) were analyzed by stepwise linear regression.

Soil depth	Activities	Regression equation	F-test	P
0–20 cm	*MF*-Cd	Y = 3.257 × 10^–19^ + 0.631 (CAR-Cd) − 0.083 (Eh) + 0.414 (EXC-Cd) − 0.271 (RES-Cd) − 0.063 (Fine silt) + 0.153 (Fine sand)	172.427	< 0.001
	*MF*-Zn	Y = 5.291 × 10^–16^ + 0.734 (CAR-Zn) + 0.138 (EXC-Zn) − 0.128 (RES-Zn) − 0.077 (Eh)	795.658	< 0.001
20–40 cm	*MF*-Cd	Y = − 9.370 × 10^–16^ + 0.833 (CAR-Cd) + 0.639 (EXC-Cd) + 0.287 (pH) − 0.587 (Eh) − 0.146 (IMO-Cd) + 0.110 (Fine silt) + 0.322 SOM	179.942	< 0.001
	*MF*-Zn	Y = − 6.932 × 10^–16^ + 0.980 (CAR-Zn) + 0.236 (EXC-ZN) − 0.069 (RES-Zn) − 0.139 (Eh) + 0.098 (pH) + 0.087 (Fine silt) + 0.042 (Fine sand)	1295.381	< 0.001

### Bioaccumulation of Cd and Zn in *B. napus* L at different growth periods

**Figure 9:**
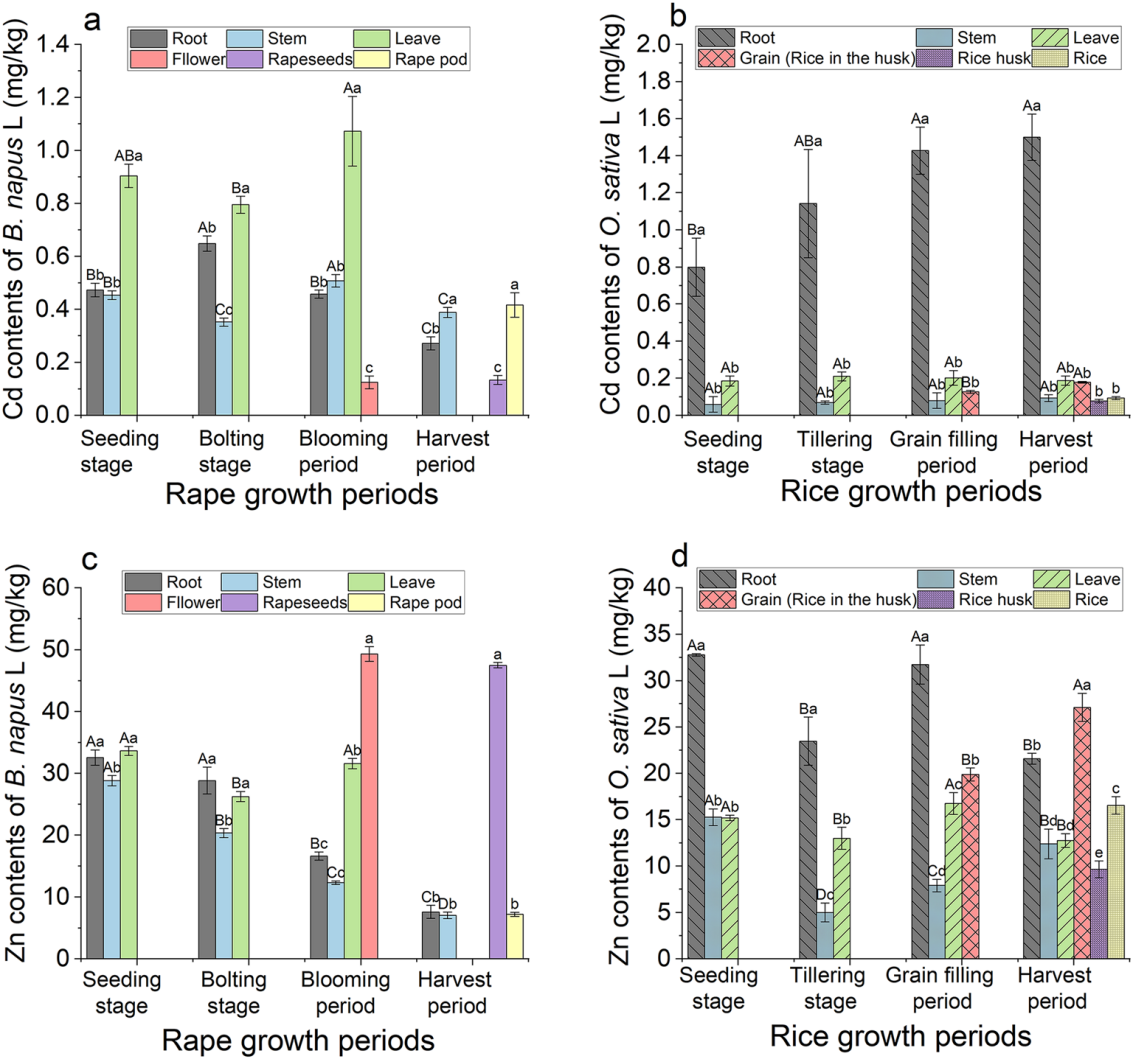
The concentration of roots, stem, leaves, bloom, rapeseed and rapeseed pod of *B. napus* L, and roots, stem, leaves, grain, rice husk and rice of *O. sativa* L for Cd (**a,b**) and Zn (**c,d**). Vertical bars represent the standard errors of the means based on three replicates, and different lower case letters indicate significantly differences between different tissues of plant at the same growth periods, and different capital letters indicate significant differences between different growth periods under the same tissues of plant (LSD test; P < 0.05).

**Figure 10:**
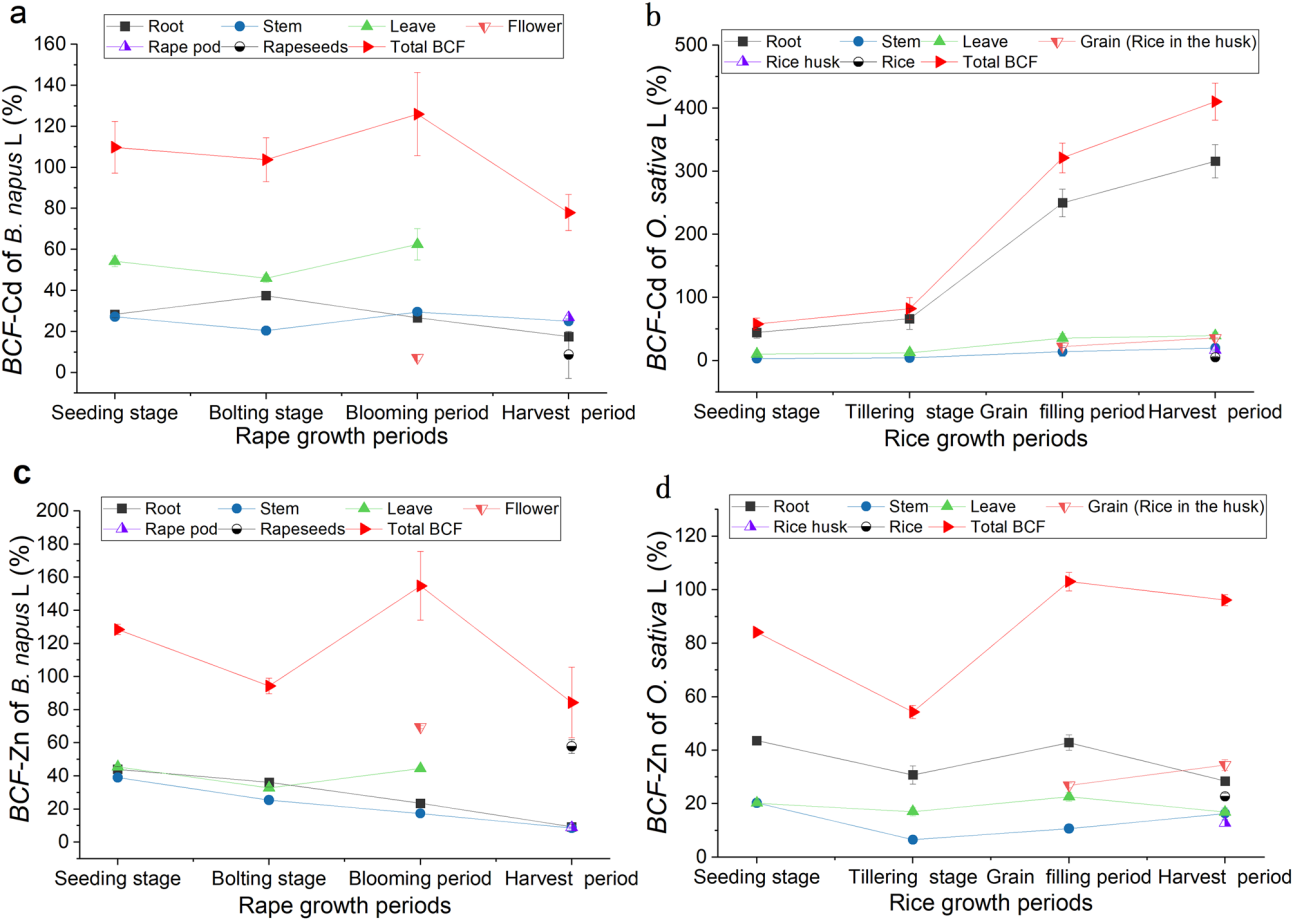
Changes in the bioconcentration factor (*BCF*) of Cd (**a**, **b**) and Zn (**c**, **d**) in different tissues of *B. napus* L and *O. sativa* L at different growth periods. Vertical bars represent the standard errors of the means based on three replicates.

From the seedling stage to the flowering stage of *B. napus* L, Cd content in leaves (0.80 mg/kg–1.07 mg/kg) (Fig. 9a) and *BCF* (45.93–62.42%) (Fig. 10a) were significantly higher than those in other parts of the plant (*P* < 0.05), and Cd was easily transferred from stems to leaves (*BTF* = 1.99–2.11) (Table 4). Therefore, leaves were the primary enrichment organs for Cd. During the harvest period, Cd contents were higher in rape pods (0.42 mg/kg) and stems (0.39 mg/kg) and *BCF* of Cd (26.8% and 25.0%, respectively) was also significantly higher than that in roots (17.5%) and rapeseed (8.6%) (*P* < 0.05) (Fig. 10a). The content of Cd in roots (0.27–0.64 mg/kg) and stems (0.35–0.51 mg/kg) increased first and then decreased during different rape planting periods. After the bolting stage, Cd transport from stems to roots gradually increased (Table 4), which resulted in higher Cd content in stems (0.51 mg/kg, 0.39 mg/kg) than in roots (0.45 mg/kg, 0.27 mg/kg) during the blooming and harvest periods (Fig. 9a). Compared with other parts, the contents of Cd in flowers (0.12 mg/kg) and rapeseed (0.13 mg/kg) were the lowest (Fig. 9a) and the *BTF* from stems to flowers (*BTF* = 0.25) was the lowest (Table 4); hence, flowers were not the primary enrichment organ for Cd. Although the transport capacity of Cd from stems to rapeseed (*BTF* = 1.07) was higher than to rape pod (*BTF* = 0.34), the content of Cd in rapeseed (0.13 mg/kg) was still significantly lower than that in rape pod (0.42 mg/kg) (Table 4). This result might be due to the short rapeseed forming period and the difficulty in transferring Cd from rape pod to rapeseed (*BTF* = 0.32).

**Table 4:** The bio-translocation factors (*BTF*) of different parts in *O. sativa* L and *B. napus* L.

Heavy metals	Parts	Rape growth periods				Parts	Rice growth periods			
		Seeding stage	Bolting stage	Blooming period	Harvest period		Seeding stage	Tillering stage	Grain filling period	Harvest period
Cd	Stem/root	0.96	0.54	1.11	1.43	Stem/root	0.07	0.06	0.06	0.06
	Leave/stem	1.99	2.26	2.11		Leave/stem	3.17	3.06	2.54	2.01
	Follower/stem			0.25		Grain/stem			1.59	1.83
	Rape pod/stem				0.34	Rice husk/stem				0.83
	Rapeseed/stem				1.07	Rice/stem				1.00
	Rapeseed/rape pod				0.32	Rice/rice husk				1.20
	Above-ground parts/root	2.87	1.77	3.72	3.46	Above-ground parts/root	0.30	0.24	0.29	0.41
Zn	Stem/root	0.89	0.71	0.74	0.92	Stem/root	0.47	0.21	0.25	0.57
	Leave/stem	1.17	1.29	2.56		Leave/stem	0.99	2.60	2.12	1.03
	Follower/stem			4.01		Grain/stem			2.52	2.11
	Rape pod/stem				6.76	Rice husk/stem				0.78
	Rapeseed/stem				1.02	Rice/stem				1.34
	Rapeseed/rape pod				6.60	Rice/rice husk				1.71
	Above-ground parts/root	1.92	1.62	5.60	8.12	Above-ground parts/root	0.93	0.77	1.40	3.59

All values are mean of three replicates (n = 3).

In contrast, the contents of Zn in flowers (49.31 mg/kg) and rapeseed (47.50 mg/kg) were significantly higher than those in other plant parts (*P* < 0.05) (Fig. 9c), and the *BCF* of flowers (69.51%) and rapeseed (57.82%) were significantly higher than those in other parts (Fig. 10c). This finding indicated that flowers and rapeseed were the primary enrichment organs for Zn in *B. napus* L. In addition to leaf drop at harvest time, the contents of Zn in leaves (26.24–33.66 mg/kg) (Fig. 9c) were also high during other growth periods and were one of the primary organs for Zn enrichment, with the exception of flowers and rapeseed. During the vegetative growth period, Zn was primarily transferred from stems to leaves (*BTF*: 1.17–2.56) and then gradually to flowers (*BTF*: 4.01) and rape pod (*BTF*: 6.76) and rape seed (*BTF*:1.02) during the blooming period (Table 4).

The variation trends in T-*BCF* of Cd (77.98–125.94%) and Zn (84.38–154.79%) in the whole plant were consistent (Fig. 10a–c) and were the greatest during the blooming period and the smallest during the harvest period. The overall variation range of Zn was greater than that of Cd. The T-*BTF* of Zn from roots to the aboveground parts was lower than that of Cd during the vegetative growth period but was significantly higher than that of Cd during the reproductive growth period (Table 4).

### Bioaccumulation of Cd and Zn in *Oryza sativa* L at different growth periods

The contents of Cd were the highest in the roots (0.8–1.5 mg/kg) during different growth periods (Fig. 9b). The content and *BCF* of Cd in the roots increased gradually with the growth of *O. sativa* L, and the *BCFs* were > 1 during the grain-filling period (249.9%) and harvest period (315.8%) (Figs. 9b, 10b). Moreover, the *BTFs* of Cd from roots to stems were only 0.06–0.07 (Table 4), thereby resulting in most of the Cd being absorbed by and then fixed in the roots, which became the primary organ for Cd enrichment. At different growth periods, both the contents (0.19–0.21 mg/kg) and *BCFs* (10.25%–39.39%) of Cd in leaves were higher than those in stems (Cd: 0.06–0.09 mg/kg; *BCF*: 3.32%–19.58%) (Figs. 9b and 10b) and the *BTFs* (2.01–3.17) from stems to leaves were all > 1 (Table 4). Hence, Cd was primarily enriched in leaves after upward transport from stems. The *BCF* of grain increased slightly from the grain-filling period to the harvest period, and the *BTFs* of Cd from stems to grain (1.59–1.83) were > 1 (Table 4). Therefore, the content of Cd in grain during the harvest period was higher than that during the grain-filling period. Additionally, the *BTF* of stem to rice husk (0.83) was lower than that to rice (1.00) (Table 4), and Cd from rice husk was continuously transferred to rice (1.21) (Table 4), which resulted in higher *BCF* of rice than that of rice husk, leaves, and stems (Fig. 10b).

Similarly, during different growth periods of *O. sativa* L, Zn contents in roots were 21.59 mg/kg–32.79 mg/kg (Fig. 9d) and *BCFs* were 28.45%–43.59% (Fig. 10d). Except for the harvest period, the contents of Zn and *BCF* in roots were higher than those in other plant parts (Figs. 9d and 10d), and the *BTFs* (0.21–0.57) of Zn from roots to stems were low (Table 4), resulting in most of the absorbed Zn being trapped in the roots (Fig. 9d). The *BTFs* (0.99–2.60) of Zn from stems to leaves were greater (Table 4), and the *BCFs* of Zn in leaves (16.82–22.63%) were higher than those in stems (6.55–20.31%) (Fig. 9d). Therefore, leaves were also an important part for Zn enrichment. The contents and *BCF* of Zn in stems decreased first and then increased, and the lowest and highest levels were observed during the grain tillering period and seedling stage, respectively. The *BCF* of grain (rice in the husk) increased significantly from the grain-filling period (26.86%) to the harvest period (34.55%) (Fig. 10d), and the *BTFs* were 2.11–2.25. This led to the contents of Zn in grain (26.21 mg/kg) being the highest during the harvest period, and the Zn content in rice (16.56 mg/kg) was higher than that in husk (9.66 mg/kg).

The bioconcentration of Cd and Zn in *O. sativa* L was seen primarily in the roots and leaves. Additionally, the T-*BCF* of Cd (57.68–410.50%) in the whole plant was much higher than that of Zn (84.11–103.05%) (Fig. 10b,d). However, the *BTF* changes for Cd and Zn in all organs were the same. The T-*BTFs* of Cd (0.24–0.41) in aboveground parts of *O. sativa* were significantly lower than those of Zn (0.77–3.59) (Table 4). Furthermore, the *BTFs* of Cd from stems to leaves (2.01–3.17) were greater than those of Zn (0.99–2.60), but the *BTFs* of Cd from roots to stems (0.06–0.07) were lower than those for Zn (0.21–0.57), and the ability of rice to transport Zn (1.34–1.71) was also higher than that of Cd (1.00–1.20). These results indicated that although the *O. sativa* L roots could absorb Cd more easily, the transport capacity of Zn from the roots to the aboveground parts of the plant was stronger.

### Influencing factors of Cd and Zn bioaccumulation during rape and rice rotation

**Figure 11:**
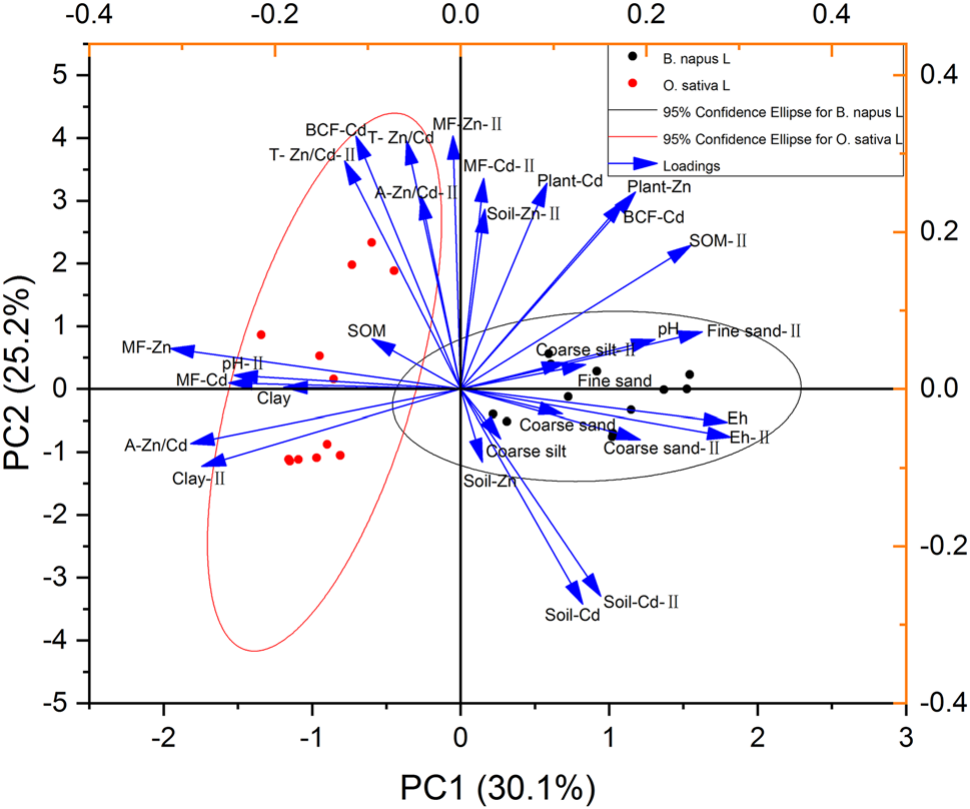
Soil physicochemical properties, Cd and Zn contents (Soil-Cd, Soil-Zn), the Cd and Zn activities (*MF*-Cd, *MF*-Zn), the ratios of Zn to Cd contents (T-Zn/Cd) and Zn to Cd activities (A-Zn/Cd) in different depths soil, Cd and Zn bioconcentration factors (*BCF*-Cd, *BCF*-Zn) and content of plants (Plant-Cd, Plant-Zn) during rape–rice rotation were analyzed by PCA. “-II” represents the deep soil.

The direct factors affecting the bioaccumulation of heavy metals in plants were their activities in soil and the abilities of plants to accumulate them. The physical and chemical properties of soil indirectly affected the uptake of heavy metals by plants as a result of affecting the activity of heavy metals in soil. PCA analysis showed that there were significant differences in physical and chemical soil properties and heavy metal migration and transformation in different plants (*O. sativa* L and *B. napus* L) and plant growth periods during crop rotation (Fig. 11). During the entire rotation process, Eh, contents and activities of Cd and Zn, and the ratio of Zn to Cd content in soil had significant effects on the total Cd bioconcentration factor (Cd-*BCF*) and total Cd contents in plants (Plant-Cd). Among them, the contents of Cd, Eh, and coarse sand in soils of different depths had negative correlations with Cd-*BCF* and Plant-Cd (Fig. 11). The content and activity of Zn, SOM, fine sand, and coarse silt in deep soil (*MF*-Zn-II, SOM-II, fine sand-II, and coarse silt-II) had positive effects on the total bioconcentration factor of Zn (Zn-*BCF*) and the total Zn contents of plants (Plant-Zn). However, clay and pH in the deep soil (Clay-II, pH-II) and clay and *MF*-Zn in the surface soil had significant negative correlations with Zn-*BCF* and Plant-Zn (Fig. 11). Additionally, the bioconcentrations and contents of Cd and Zn in plants were positively correlated (Fig. 11).

However, during the entire rape–rice rotation process, owing to differences in plant species (*O. sativa* L and *B. napus* L) and flooding conditions (alternating drought and flood), the influencing factors of heavy metal bioaccumulation of the two crops were different. Therefore, further stepwise linear regression simulation analysis was conducted on the influencing factors of *O. sativa* L, and *B. napus* L bioaccumulation (Table 5). These results indicated that deep soil had more significant effects on Cd bioaccumulation, whereas the effects of surface soil on Zn bioaccumulation was more significant during rape growth.

**Table 5:** The influencing factors of *O. sativa* L and *B. napus* L bioaccumulation were analyzed by stepwise linear regression.

Plants		Regression equation	F-test	P
*B. napus* L	Plant-Cd	Y = 1.195 × 10^–16^ − 0.972 (T-Zn/Cd) + 0.739 (SOM-II) + 0.498 (T-Zn/Cd-II)	26.928	< 0.001
	*BCF*-Cd	Y = 8.688 × 10^–16^ − 1.104 (T-Zn/Cd) + 0.668 (SOM-II) + 0.617 (T-Zn/Cd-II) + 0.371 (pH) − 0.293 (A-Zn/Cd-II)	65.345	< 0.001
	Plant-Zn	Y = 7.137 × 10^–16^ − 0.871 (Soil-Zn) − 0.118 (Coarse sand) − 0.148 (Clay) + 0.059 (SOM) + 0.069 (SOM-II) − 0.166 (A-Zn/Cd) + 0.022 (pH)	2540.24	< 0.001
	*BCF*-Zn	Y = 4.190 × 10^–16^ − 0.806 (Soil-Zn) − 0.213 (*MF*-Zn) + 0.122 (Soil-Cd-II) − 0.106 (Coarse sand)	160.206	< 0.001
*O. sativa* L	Plant-Cd	Y = 3.824 × 10^−17^ + 1.771 (*BCF*-Cd) − 1.385 (*MF*-Zn) − 0.539 (Fine sand-II) + 0.635 (Soil-Cd-II) − 0.397 (SOM-II) + 0.156 (Soil-Zn-II)	84.046	< 0.001
	*BCF-*Cd	Y = -3.062 × 10^−16^ − 0.575 (Soil-Cd) + 0.281 (*MF*-Zn-II) + 0.216 (T-Zn/Cd)	60.257	< 0.001
	Plant-Zn	Y = − 2.154 × 10^−16^ + 1.015 (*BCF*-Zn) + 0.090 (Soil-Zn) + 0.050 (pH-II) + 0.081 (SOM-II)	3068.31	< 0.001
	*BCF-*Zn	Y = − 2.106 × 10^−15^ + 0.608 (SOM-II) − 0.728 (Soil-Zn) + 0.230 (Fine sand-II) − 0.161 (Soil-Cd-II)	41.76	< 0.001

“- II” represents the deep soil.

The SOM-II and ratio of Zn to Cd content in deep soil (T-Zn/Cd-II) had significant positive effects on Plant-Cd and Cd-*BCF* of *B. napus* L, whereas the ratio of Zn to Cd content in surface soil (T-Zn/Cd) had a significant negative effect (Table 5). Additionally, the ratio of Zn to Cd activity in deep soil (A-Zn/Cd-II) had a significant negative correlation with Cd-*BCF* of *B. napus* L (Table 5). For Soil-Zn, coarse sand had significant negative effects on Plant-Zn and Zn-*BCF* of *B. napus* L. Clay and the ratio of Zn to Cd activity in surface soil (A-Zn/Cd) had significant negative effects on Plant-Zn, whereas SOM and pH increased Plant-Zn in *B. napus* L. It is noteworthy that Soil-Cd-II promoted Zn-*BCF* of *B. napus* L, whereas *MF*-Zn was significantly negatively correlated with Zn-*BCF* (Table 5). Moreover, *MF*-Cd had no significant effect on the Cd bioaccumulation of *B. napus* L, but the Zn/Cd ratio in soil had a significant effect (Table 5).

During the process of *O. sativa* L growth, the influencing factors of Plant-Cd and Cd-*BCF* were somewhat different. The Plant-Cd was not only positively correlated with Cd-*BCF*, Soil-Cd-II, and Soil-Zn-II but also negatively correlated with *MF*-Zn and fine sand-II. While the Cd-*BCF* of *O. sativa* L was significantly negatively correlated with Soil-Cd, it was significantly positively correlated with *MF*-Zn-II and T-Zn/Cd (Table 5).

Zn-*BCF* of *O. sativa* L, Soil-Zn-II, pH-II, and SOM-II had significant positive effects on Plant-Zn of *O. sativa* L (Table 5). The Zn-*BCF* of *O. sativa* L was significantly positively correlated with SOM-II and fine sand-II but negatively correlated with Soil-Zn and Soil-Cd-II (Table 5).

The Zn/Cd ratio in soil was found to have significant effects on Cd-*BCF* of *B. napus* L and *O. sativa* L. Furthermore, the Cd content in soil was also significantly correlated with Zn-*BCF* of *B. napus* L and *O. sativa* L, but the mechanism was affected by soil depth (Table 5).

## Discussion

### Bioaccumulation capacity of Cd and Zn in the soil by *B. napus* L and *O. sativa* L was different

The absorption and transport pathways of heavy metals in plants include active absorption and passive absorption by roots, transfer from stems to other parts of the plant. Heavy metals in stem, leaves and inflorescence are reactivated and transported into grain through phloem during flowering to seed maturity stage^40^. Additionally, plants transport Cd directly into seeds through xylem^41,42^. Therefore, the enrichment capacity of heavy metals was seen to increase during the grain development period. In this study, it was also confirmed that Cd and Zn absorbed by the roots of *B. napus L* and *O. sativa L* before the reproductive stage were mostly transported to the leaves and then transferred to the seeds during the reproductive stage. However, the migration and accumulation of elements varies in different plants. In rapeseed, the transport capacity of Cd was seen to be higher than that of Zn during the vegetative growth stage but lower than that during the reproductive stage (Table 4). Flowers and grains were the main enrichment organs for Zn, whereas their Cd content was the lowest (Fig. 9). Moreover, Zn was more easily translocated to rice grains than Cd (Table 4) probably because Zn is an important component of many enzymes and can promote the synthesis of carbohydrates and proteins. Therefore, the translocation of Zn to flowers and grains during the reproductive period is beneficial and increases the protein content of grains^43,44^. Furthermore, Zn enrichment may inhibit the transfer of Cd into seeds^29,45,46^.

This study found that although Cd was more easily absorbed by *O. sativa* L roots, Zn was more easily transported upward (Table 4). Cd and Zn enrichment capacities of *B. napus L* and *O. sativa L* were different. The enrichment ability of Cd in *O. sativa L* was higher than that in *B. napus L* but was the opposite for Zn. Conversely, the enrichment capacity of Zn in *B. napus L* was higher than that of Cd but was the opposite in *O. sativa L* (Fig. 9). It has been confirmed that heavy metal accumulation capacity varies widely among different varieties and species and that it is affected by the levels of heavy metals in the soil^32^. This study also found that the *BCF* of Cd and Zn in *B. napus* L was not the key factor affecting the contents of Cd and Zn in plants. The physical and chemical soil properties and the Zn/Cd ratio were the primary factors affecting the content in plants (Table 5). This result contrasts with the contents of Cd and Zn in *O. sativa L*, which were closely related to their *BCFs* (Table 5), and it is consistent with the findings of previous studies^39,47^. In addition to different metal properties, the affinity of different plants and the influence of physical and chemical soil properties on Cd and Zn were seen to be related. Some studies have found that *O. sativa L* is a unique crop with strong Cd accumulation capacity and significant Zn exclusion^48,49^. Interestingly, the rhizosphere exudates of *B. napus* L activate soil available cadmium to varying degrees, which have an impact on the cadmium content of rice grains in subsequent plantings^50^. In contrast, a reducing environment with low REDOX potential was formed due to the flooding environment in the paddy field, and hydrogen sulfide produced by the anaerobic decomposition of organic matter forms cadmium sulfide precipitates with Cd in the soil, thus reducing its availability^26^.

### Soil-Zn/Cd ratio was closely related to the bioenrichment of Cd and Zn in rape and rice during crop rotation

In soil systems, the interactions between heavy metals primarily occur at the substrate, absorption, and target levels, and there is site competition for heavy metals at all three levels^51^. Cd stress leads to severe Cd exposure and Zn deficiency in rice grains^44^, and high Cd/Zn ratios in rice could exacerbate the potential biotoxicity of Cd^23^. However, several studies have shown that the Cd/Zn ratio of crops inhibits Cd absorption to a certain extent^31,32,35^. The possible interaction between Cd and Zn in soil adsorption and plant absorption^32,42,44,45,52^ is also one of the reasons for the difference in Cd and Zn enrichment between *B. napus L* and *O. sativa.* In this study, the bioenrichment of Cd in rape was closely related to the Zn/Cd ratio in soil at different depths. The Zn/Cd ratio in topsoil inhibited the absorption of Cd in rape, but promoted the enrichment of Cd in rice (Table 5). This may be related to the significant increase of Zn/Cd in soil during rice planting period. However, the correlation between Zn/Cd ratio in soil and Zn bioenrichment in rapeseed and rice was low. When Cd toxicity predominates, the presence of Zn antagonizes cadmium and reduces the toxicity of Cd to organisms. On the contrary, when Zn toxicity predominates, the presence of Cd has a synergistic effect with zinc and increases the toxicity of Zn to organisms^35,53^.

### Effects of soil physicochemical properties on the bioavailability of Cd and Zn during rice–rape rotation

#### Soil Eh

The change in Eh reflected the redox state of soil. Soil Eh decreased gradually from rape planting season to rice planting season and was in a reducing state. This observation was consistent with the results of previous studies where Eh was noted to decrease during the flooding process and increase during the drying process^54,55^. The Eh of surface soil should be higher owing to better soil aeration. However, good aeration is also more conducive to the growth of aerobic microorganisms, which consume oxygen during the growth process and in turn reduce the soil Eh^36^. This may be the reason for the Eh of surface soil being slightly lower than that of deeper soil during the rape planting period.

In this study, Eh did not directly affect the bioavailability of Cd and Zn (Table 4) but did so indirectly by affecting the activities of these heavy metals in the soil. The degree of toxicity of the chemical forms of heavy metals is EXC > CAR > IMO > OM > RES^23^, and this study found that there was a significant positive correlation between soil Eh and the RES of heavy metals (*P* < 0.01) (Table 2). In surface soil, the contents of CAR-Cd, CAR-Zn, EXC-Zn, and IMO-Zn increased significantly with the decrease in Eh, whereas the contents of OM-Zn decreased significantly. In deep soil, EXC-Zn and OM-Cd increased with the decrease in Eh (Fig. 6, Table 2). Therefore, Eh reduction was found to be one of the causes for heavy metal activation (Fig. 8a,b,d). Chuan et al.^56^ also observed that when the Eh of soil solution decreased, the concentration of exchangeable Cd increased. Therefore, Eh determines the solubility change of heavy metals in the process of rice field–upland field rotation^25,57^.

In soil, redox substances, such as iron and manganese oxides and sulfides, indirectly affect the solubility and morphology of heavy metals in the soil^28–30^. When Eh decreases (such as in flooded environments), reductive substances increase in soil, a large number of iron and manganese oxides are reduced and dissolved, and previously stable adsorbed metal ions are released into soil solution^27^. Moreover, reduced Fe^2+^ and Mn^2+^ may compete with heavy metal ions and lead to their release^27^, thus enhancing the migration of heavy metal ions. Similar results were observed in this study where the percentages of residual fractions of Cd and Zn decreased with the decrease in Eh (Fig. 6) (Table 2) and the activities increased (Fig. 8a–b). However, the correlations between Eh and the IMO of metals in different soil depths were different. This observation may be related to the different redox environments due to the degree of change resulting from surface soil flooding and drying more than did deeper soil and the difference in pH values and SOM (Fig. 3). It is also possible that the adsorption capacity of different iron oxides to heavy metals is different (the adsorption capacities were in the following order: amorphous iron oxides > maghemites > lepidocrocite > goethite)^58,59^. With the decrease in Eh + pH, iron oxides change from amorphous forms with strong adsorption ability to microcrystalline forms with weak adsorption ability^60^. Therefore, Eh can change the speciation of iron and manganese oxides, and thus, affect the environmental behavior of heavy metals.

#### Soil pH

The effect of Eh on the activity of heavy metals is primarily through the influence of soil pH or through the interaction with pH change^61^. Soil pH changes in response to the change in Eh, and different H^+^ activities directly affect the chemical species and the migration and transformation processes of heavy metals^62^. In this study, the carbonate binding fractions of Cd and Zn were the main factors that enhanced their activity, whereas the residual fractions were the main forms that limited their activity (Table 3). pH had negative correlations with Cd and Zn in the bonded fraction of carbonate and the oxidized fraction of iron–manganese and significant positive correlations with Cd and Zn in the residue fraction (Table 2). These correlations were closely related to the fact that the soil in the test area was neutral to alkaline (pH = 6.9–8.11) (Fig. 3a), which is a typical calcareous soil associated with carbonate rock. In general, most heavy metals in calcareous soil with pH > 7 are converted into carbonate fractions, and the increase in pH is conducive to the formation of carbonate^63^. However, some studies have found that Cd was likely to be hydrolyzed into Cd(OH)_2_ or Cd(OH)^+^ at pH 6.0–8.0^64^. In waterlogged environment, CdHCO_3_ and Cd(OH)_2_CO_3_ can be formed by hydrolysis of metal elements in the carbonate binding state, resulting in alkaline water and increased pH. In addition, the change of Eh is the main factor affecting the activity of heavy metals during crop rotation, and pH value is affected by Eh. It was found that soil pH tended to be neutral after flooding. Wang et al.^65^ showed that when pH was 6.0–8.5, it continued to decrease as Eh decreased because of the decomposition of organic matter into a variety of small organic acids and carbon dioxide. In this study, there was a slight change in pH under flooding condition. It may be that a large amount of carbonate hydrolysis in the soil where carbonate rocks developed led to the increase of pH, which played a buffering role in pH change under flooding condition^66^. Therefore, in this study, owing to the complexity of soil composition, there is not a single positive/negative correlation between Eh and pH^67^, and under the comprehensive influence of complex changes in soil chemical environment, pH changes are negatively correlated with Cd and Zn in the bonded fraction of carbonate. However, the specific mechanism of the effect of pH value on the change of carbonate in the dry–wet alternating environment needs further investigation.

In addition, Cd(OH)^+^ and Zn(OH)^+^ formed by the hydrolysis of Cd and Zn in the bonded fraction of carbonate have strong affinity and can be adsorbed on the surface and lattice of soil minerals via electrostatic adsorption, ion exchange, and hydrogen bonding^68^. In alkaline environments, iron and manganese oxides are mostly negatively charged, and the adsorption of heavy metals increases rapidly with the increase in pH^69^. Therefore, heavy metals primarily exist in the form of compounds in alkaline soil, which may be the reason for the low activity of heavy metals in soil observed in this study (*MF*-Cd: 6.51–12.31%; *MF*-Zn: 0.48–1.90%).

It is noteworthy that in this study, pH had no significant effect on Cd and Zn activities in surface soil but had a positive and significant effect in deep soil (Table 3). This observation may be because the pH of deep soil was more sensitive to the influence of heavy metal speciation than that of surface soil, and as can be inferred from Fig. 3a, the pH increased with soil depth. This was consistent with the distribution rule of pH values in different soil depths^70,71^.

#### Soil organic matters

In addition to pH, Eh may also affect the composition of organic matter^72^. SOM possesses complex organic functional groups that can adsorb, complexate, or chelate metal ions^73^. Moreover, SOM has strong reducibility, which can reduce highly-charged heavy metals and alter their toxicity^74,75^. In this study, the variation range of organic matter in the surface soil was low, whereas the variation degree in deep soil was more obvious, especially after rapeseed harvest, which was significantly reduced during the flooding process (Fig. 3c). When flooded in deep soil in an anaerobic environment for a long time, macromolecular organic matter easily acts as an electron acceptor through microbial reduction into dissolved organic matter (DOM)^76^. Meanwhile, metals bound to organic matter are released as the organic matter decomposes and then combine with DOM^77^, thereby resulting in increased metal concentrations in soil solutions in overlying water and increased availability^78,79^. This is one of the main reasons for the observed increase in soil metal (Cd and Zn) activities during rice planting season and also for the bioaccumulation of metals in *B. napus* L and *O. sativa* L being significantly affected by SOM (Table 5). Zhao et al. also showed that organic matter increases the bioavailability of Cd in rice^80^. The SOM increase in deep soil during the harvest stage may be due to the reduced nutrient requirements of plants and the increased amount of organic matter in deep soil due to senescent roots. Accordingly, soil DOM content also increases and competes with heavy metals for adsorption sites on soil surfaces, thus reducing the adsorption capacity of soil for heavy metals and increasing the exchangeable metals ions. Consequently, the activities of Cd and Zn in deep soil increased to varying degrees (Fig. 8a). Additionally, drainage during the later stages of *O. sativa L* growth altered the redox environment of the underlying soil, and the mineralization and decomposition of organic matter also released metal ions.

#### Soil particle sizes

Soil texture has a major impact on soil adsorption and affects the distribution and bioavailability of heavy metals^81^. In general, smaller soil particles have better adsorption properties and, therefore, higher heavy metal concentrations^82,83^. This study reached a similar conclusion that heavy metals were primarily concentrated in particles sized 0.05–0.25 mm. During the rice planting period, the proportional increase in small soil particles (Fig. 2) and the percentage of clay (< 0.01 mm) in the soil were negatively correlated with the bioavailability of heavy metals (Fig. 11). This finding indicates that water caused certain mechanical dispersion forces that affected the soil particles, reduced the soil particle size, increased the adsorption of heavy metals, and reduced the availability of Cd and Zn. These effects were far lower than the effective enhancement of heavy metals by redox environment changes caused by water logging. Hence, the activity of heavy metals continued to increase after waterlogging (Fig. 8). Soil particle size generally does not directly affect the chemical species of heavy metals but affects the concentration of heavy metals in the soil solution through their absorption–desorption by soil particle size components. Thus, the combination, complexation, or chelation of heavy metals with organic substances, clay minerals, and other organic and inorganic colloids is affected^82,83^. Consequently, the distribution proportions of heavy metals in soil grains affect their toxicity^84^.

In conclusion, the frequent interaction of soil phases makes the activity of each relative heavy metal extremely complicated, and the difference of heavy metal enrichment of rotating plants leads to the difficulty of controlling heavy metals under rotation mode. Eh is an important variable affecting the migration of heavy metals in soil of rotation system, while Fe, Mn, and sulfide in soil components are essential media, and soil microorganisms are also crucial to the chemical form of heavy metals. Therefore, the above factors should be considered in the study of heavy metal release in soil with Eh fluctuations, so as to better understand and predict the dynamic change mechanism of heavy metals under the influence of complex alternating dry and wet effects. In addition, the dynamic changes of heavy metals in the rotation system under spatial heterogeneity and time scale, as well as the quantitative relationship between soil environment and heavy metal activity in the process of rapeseed–rice rotation will be further studied.

## Conclusion

This study found that the physical and chemical properties of soil and heavy metal migration and transformation processes were significantly different during the growth periods of different plants (*B. napus* L and *O. sativa* L). Soil-Cd content varied greatly at different depths, and Zn content in surface soil fluctuated greatly compared with that in deep soil. Due to the change of soil physical and chemical properties, the chemical species and activities of Cd and Zn changed significantly and were activated obviously during the rice growth season, in which Zn chemical species and activity were more easily affected. In the rotation process, the enrichment ability of Cd in *O. sativa* L was stronger, while that of Zn in *B. napus* L was stronger, and the transport characteristics of Cd and Zn in different organs of the two crops were also different. Cd and Zn contents in the two crops were mainly affected by the physical and chemical properties of the soil and Soil-Zn/Cd ratio, whereas that of *O. sativa* L was also closely related to the enrichment ability. Zn and Cd exhibited certain interactions in soil–plant systems, especially in deep soil. Eh was the main factor affecting the chemical properties and activities of soil heavy metals, but it was not a direct factor affecting the bioenrichment of *O. sativa* L and *B. napus* L. However, the changes in soil SOM, pH, and grain size affected the bioenrichment ability of *B. napus* L. In conclusion, alternating dry and wet cropping changed the activities of Cd and Zn, soil properties, and heavy metal activity, which differed during different growing stages of crops. The absorption capacity of different crops toward different heavy metals also varied. Therefore, corresponding mitigation actions should be taken according to the specific soil environment and plant characteristics involved in the treatment process.

## Data Availability

The datasets generated during and/or analyzed during the current study are available from the corresponding authors on a reasonable request.
